# The role of transmembrane proton transport rates in mild mitochondrial uncoupling by arylamide substituted fatty acids

**DOI:** 10.1039/d5sc06530e

**Published:** 2025-12-02

**Authors:** Ethan Pacchini, Daniel A. McNaughton, Aaron Pye, Katie A. Wilson, Philip A. Gale, Tristan Rawling

**Affiliations:** a School of Mathematical and Physical Sciences, Faculty of Science, University of Technology Sydney Sydney NSW 2007 Australia tristan.rawling@uts.edu.au; b Department of Biochemistry, Memorial University of Newfoundland St. John's NL A1C 5S7 Canada

## Abstract

Mitochondrial uncoupling by small molecule protonophores is a promising therapeutic strategy for leading diseases including obesity, diabetes and cancer, however the clinical potential of these agents is complicated by their associated toxicity. Protonophores that exclusively produce mild uncoupling can circumvent toxicity concerns, but these compounds or a framework to guide their design is currently lacking. In this study, we prepared a series of atypical arylamide-substituted fatty acid protonophores and found that specific aromatic substitution patterns can fine-tune their uncoupling activity. Notably, 3,4-disubstituted arylamides were found to increase cellular respiration and partially depolarise mitochondria without compromising ATP production or cell viability. These are hallmarks of mild uncoupling. In contrast, 3,5-disubstituted arylamides mimicked the full uncoupling effects of the classical uncouplers DNP and CCCP. Mechanistic studies revealed a diminished capacity for the 3,4-disubstituted arylamides to self-assemble into membrane permeable dimers in the rate limiting step of the protonophoric cycle. This translated into overall slower rates of transmembrane proton transport, and may account for their mild uncoupling activity. This work represents the first exploration of how proton transport rates influence mitochondrial uncoupling and provides a new conceptual framework for the rational design of mild uncouplers.

## Abstract 2

Arylamide fatty acids are mild mitochondrial uncouplers that partially depolarise mitochondria without compromising ATP synthesis. Mild activity results from diminished proton transport rates, providing a new basis for design of safer uncouplers.

## Graphical abstract

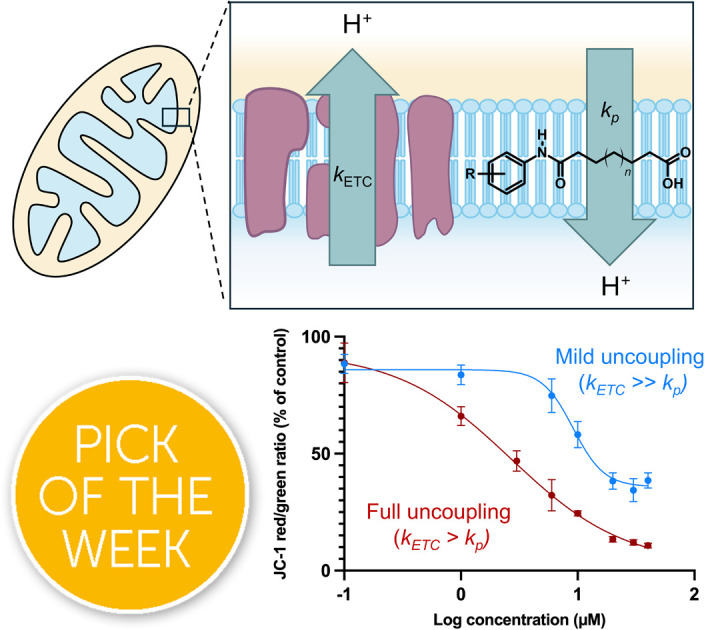

## Introduction

Mitochondria play critical roles in cell function, including converting energy from nutrients into adenosine triphosphate (ATP) through oxidative phosphorylation (OxPhos).^1^ In this process, nutrients are oxidised in the mitochondrial matrix to produce high-energy electron carrier molecules NADH and FADH_2_, which are then fed into the electron transport chain (ETC), a series of proteins embedded in the mitochondrial inner membrane (MIM). As electrons flow through the ETC protons are pumped from the matrix and into the intermembrane space to produce a proton gradient (ΔpH) across the MIM. The resulting membrane potential (Δ*Ψ*_m_) generates a proton motive force (PMF) that drives the flow of protons back into the matrix through ATP-synthase, catalysing the conversion of adenosine diphosphate (ADP) into ATP. Thus, nutrient oxidation and ATP synthesis are coupled by mitochondrial proton gradients.

**Fig. 1:**
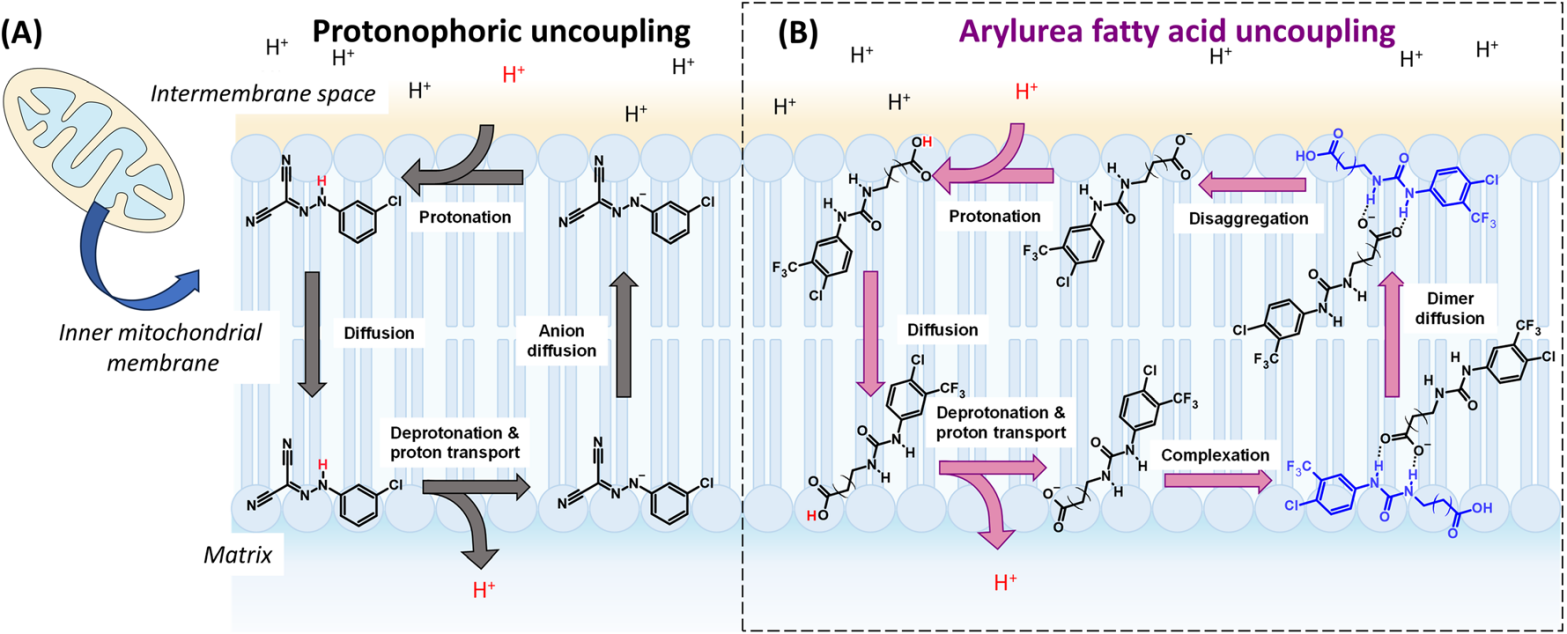
Transmembrane proton transport and mitochondrial uncoupling by classical protonophores and arylurea substituted fatty acids. (A) Mechanism of proton transport and uncoupling by classical uncoupler CCCP. In the rate-limiting anion diffusion step, negative charge is delocalised across the extended π-system to promote permeation of the anion across the MIM and further cycles of proton transport. (B) The mechanism of proton transport by arylurea fatty acids. These agents traverse the MIM *via* self-assembly into membrane-permeable dimers where the urea group functions as an anion transporter to diffuse the charge of the carboxylate group and facilitate passage across the MIM.

Mitochondrial uncoupling occurs when protons leak across the MIM and back into the matrix without passing through ATP-synthase, leading to dissipation of the PMF and inhibition of ATP synthesis. Mitochondrial uncoupling can be induced by protonophores such as CCCP that transport protons across the MIM *via* repeated cycles of de/protonation of the protonophore's acidic functional group (see Fig. 1A).^2,3^ The rate-determining step of the protonophoric cycle is the permeation of the deprotonated anionic uncoupler across the hydrophobic core of the MIM. For this reason, protonophores typically possess extended π-systems that allow for the delocalisation of negative charge and formation of lipophilic and membrane-permeable anions.^4^

The therapeutic potential of protonophoric uncouplers was first recognised in the 1930s with 2,4-dinitrophenol (DNP), which reduced the efficiency of OxPhos and increased nutrient metabolism in humans, inducing rapid weight-loss.^5,6^ Despite its efficacy DNP was pulled from the market due to serious adverse side effects, including fatal hyperthermia that arose as the energy stored in mitochondrial proton gradients was diverted from ATP production and instead released as heat.^7^ Since then uncouplers have been viewed as too toxic for human use but that perspective is changing as recent studies have revealed beneficial effects of uncoupling in various disease models, including obesity, diabetes, cancer and various forms of neurodegeneration.^1,8–14^ This has driven research into the development of safe and well-tolerated uncouplers, such self-limiting DNP-triphenylphosphine conjugates (mitoDNP)^15,16^ and DNP prodrugs that provide controlled delivery of DNP *in vivo*.^2,17^ Indeed the DNP prodrug HU6 is currently progressing through phase II clinical trials (NCT05284617). Novel protonophore scaffolds such as BAM15 and related oxidazoles have also been developed.^12,13,18^ These agents have well-characterised uncoupling activity and promote weight loss in mouse models without apparent toxic side effects. The favourable safety profile of these compounds is attributed to their capacity to selectively depolarise the MIM and not plasma membranes.^19^ The uncouplers OPC-163493 and Ppc-1 are also reported as safe, although the mechanism/s underlying their safety has not been identified.^20,21^

A compelling alternative approach is mild uncoupling, which is characterised by a partial reduction of the proton gradient that weakly stimulates respiration without compromising ATP synthesis.^2,22–28^ As mild uncouplers do not extensively depolarise mitochondria they lack capacity to induce hyperthermia and other side effects associated with full uncoupling, making them attractive candidates for long-term therapeutic used. Indeed, mild uncoupling is an endogenous process mediated by uncoupling proteins.^29^ Mild uncoupling can be produced pharmacologically using low concentrations of full uncouplers^25^ including DNP^27,30^ and carbonyl cyanide-*p*-(trifluoromethoxy)phenylhydrazone (FCCP).^31,32^ This approach has produced promising results in experimental models of disease but still carries the inherent toxicity risks of full uncouplers. Thus, identification of pharmaceutical agents that exclusively produce mild uncoupling is important. Lipophilic cations have the potential to act as mild uncouplers as they accumulate in polarised mitochondria and disperse into the cell cytosol as depolarisation occurs, providing a self-limiting mechanism. To this end, triphenylphosphonium derivatives^3,33^ and SkQ family of lipophilic cations have been explored as mild uncouplers with several compounds producing promising weight-loss in animal models of obesity.^34–36^ As lipophilic cations lack the chemical features of conventional protonophores, their uncoupling actions occur *via* a different mechanism/s that may involve interaction with free fatty acids,^24^ or *via* inhibition of ATP-synthase.^28^ To date no protonophores that exclusively produce mild uncoupling have been discovered.

Recently, we reported the development of arylurea-substituted fatty acids (termed arylureas) as a new class of mitochondrial uncoupler.^37^ Unlike typical protonophores, these agents utilise an anion transport mechanism to complete the rate-limiting step of the protonophoric cycle (Fig. 1B). Following deprotonation in the matrix, the arylureas self-assemble into dimers *via* intermolecular hydrogen bonding between the carboxylate group of one molecule the urea group of another. Delocalisation of the carboxylate charge across the urea group renders the complex sufficiently lipophilic to permeate the MIM and carry out repeated cycles of proton transport. The arylureas act as full uncouplers in MBA-MD-231 breast cancer cells and induce cell death by inhibiting ATP production and inducing mitochondrial dysfunction.^37,38^ In this work, we prepared a related series of arylamide-substituted fatty acids (termed arylamides) and assessed their mitochondrial actions in MDA-MB-231 cells. All arylamides were active uncouplers, however a subset of arylamides produced cellular effects that were consistent with mild mitochondrial uncoupling. Vesicle-based assays revealed these arylamides transport protons across lipid bilayers at lower rates than those that produce full uncoupling, indicating that manipulation of proton transport rates can be used to develop mild mitochondrial uncouplers.

## Results & discussion

### Compound library and synthesis

The arylamide fatty acids were designed as structural analogues of the previously reported aryl urea fatty acids. The aromatic rings of the arylureas were substituted with lipophilic electron-withdrawing groups, as these substituents promote formation of lipophilic and membrane-permeable dimers during the protonophoric cycle (see Fig. 1B) and optimise uncoupling activity.^37,38^ Specifically, the electron-withdrawing substituents enhanced the carboxylate affinity of urea N-Hs hydrogen to favour dimer formation and their lipophilicity improves the membrane permeability of the resulting dimers. Thus, in the arylamide series, chloro- and trifluoromethyl substituents were introduced in 3,5- and 3,4- substitution patterns. The arylureas possessed a 16-carbon fatty acid chain. In the arylamide series, the chain was extended to 18 carbons to match the overall length of their urea counterparts.

**Scheme 1:**
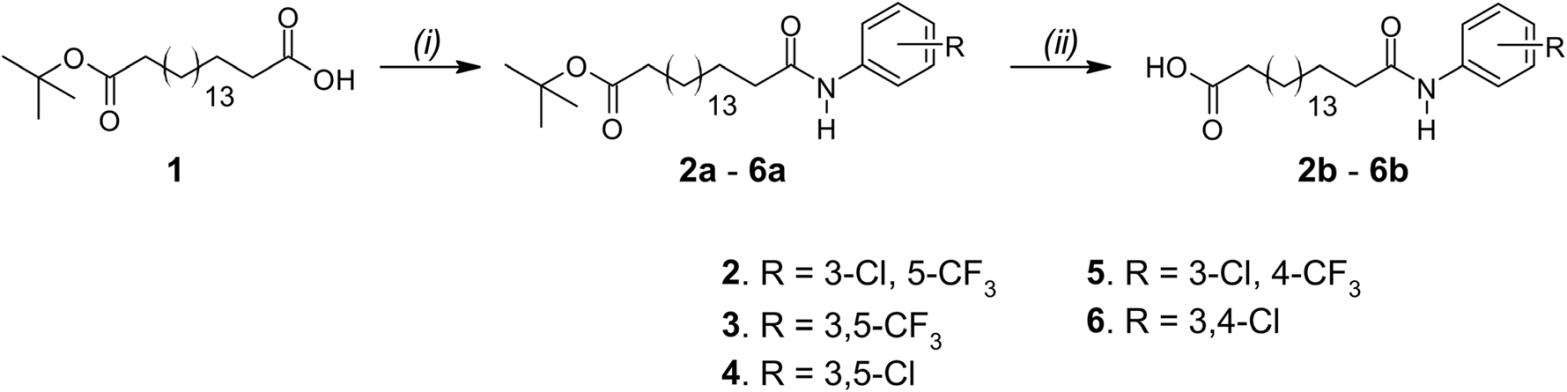
Synthesis of arylamides 2b–6b. (i) COMU, Et_3_N, substituted aniline, DMF; (ii) TFA, CH_2_Cl_2_.

Synthesis of arylamide fatty acids was achieved in 2 steps, starting from *tert*-butyl ester-protected octadecanedioic acid (Scheme 1). Compound 1 was reacted with appropriately substituted anilines and COMU in the presence of triethylamine (Et_3_N) under anhydrous conditions to afford the arylamides 2a–6a. The *tert*-butyl ester protection group was removed through TFA-catalysed hydrolysis and liquid-liquid extractions with NaOH, water and HCl afforded arylamide fatty acids 2b–6b in excellent yields (88–95%).

### Effects of arylamides on MDA-MB-231 cell viability

**Fig. 2:**
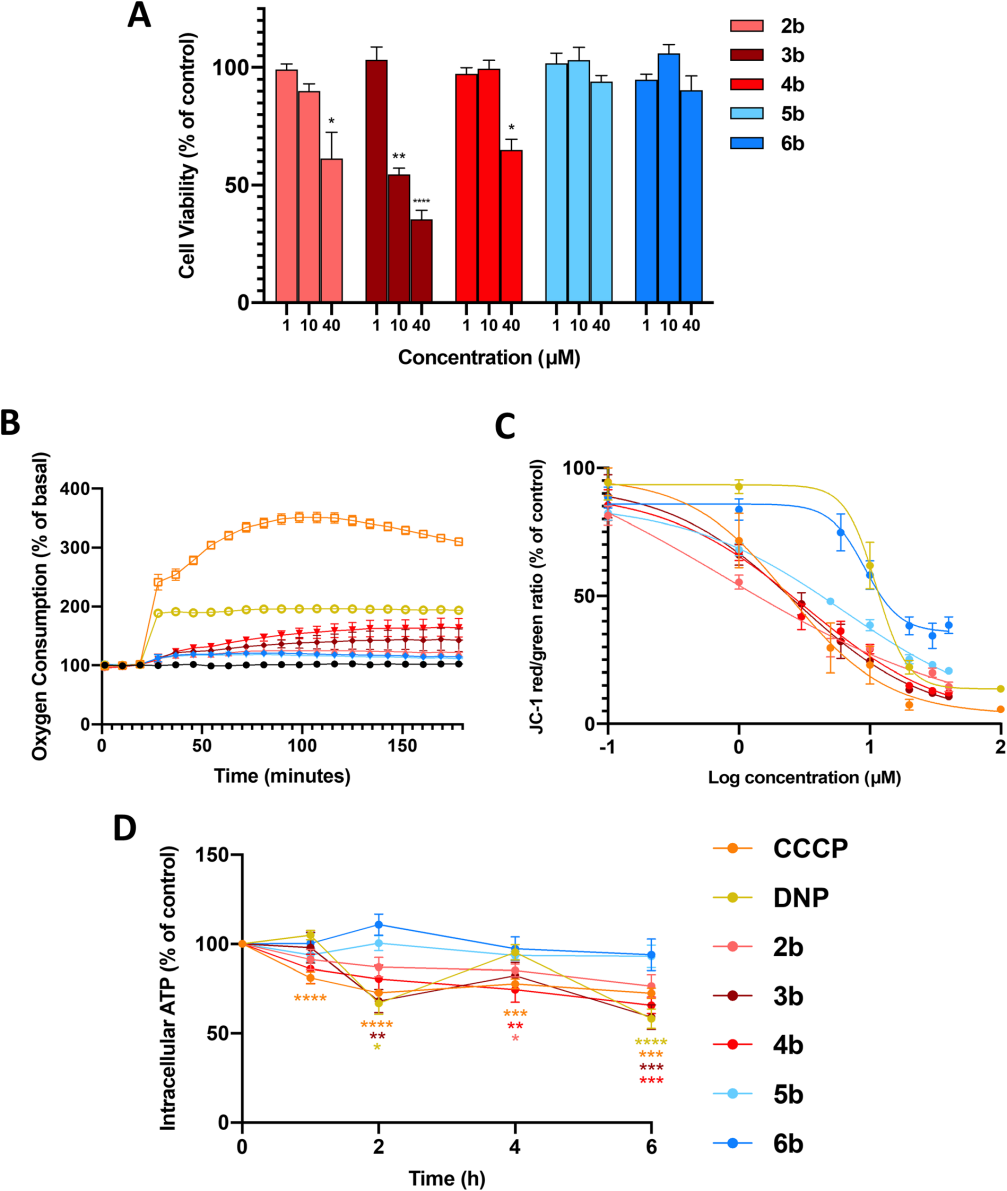
*In vitro* effects of arylamides 2b–6b, DNP and CCCP in MDA-MB-231 cells. (A) Viability of MDA-MB-231 breast cancer cells treated with arylamides 2b–6b (24 h). Data represents the mean ± SEM of 3 independent experiments. Difference from DMSO control: (*) *P* < 0.05, (**) *P* < 0.001, (****) *P* < 0.0001. (B) OCR of MDA-MB-231 breast cancer cells when treated with arylamides 2b–6b at their maximal solubility limit (40 µM) as well as CCCP (5 µM) and DNP (20 µM) over 3 h. Data represents mean ± SEM of 3 independent experiments. (C) Dose–response curves showing the effects of arylamides 2b–6b (1 h) on JC-1 red/green fluorescence ratios. Data represents the mean ± SEM of 3 independent experiments. (D) Intracellular ATP levels in MDA-MB-231 cells treated with amides 2b–6b, DNP and CCCP at 20 µM for 1–6 h. Data represents the mean ± SEM of 3 independent experiments. Difference from time-matched DMSO control: (*) *P* < 0.05, (**) *P* < 0.01, (***) *P* < 0.001, (****) *P* < 0.0001.

Full mitochondrial uncouplers such as CCCP and DNP reduce the viability of cancer cells,^16^ so we first examined the effects of arylamides 2b–6b (1, 10 and 40 µM, 24 h) on the viability of MDA-MB-231 breast cancer cells using the MTS assays. In these assays, and all subsequent cell-based assays, the maximum test concentration of each arylamide was 40 µM, as the compounds precipitated out of the cell media at higher concentrations. As shown in Fig. 2A arylamide 3b was the most active in the series and reduced cell viability to 54.7 ± 4.5% (*P* < 0.001) and 35.4 ± 6.5% (*P* < 0.0001) of control at 10 and 40 µM, respectively. 2b and 4b also significantly reduced cell viability at 40 µM, while 5b and 6b showed no activity at all test concentrations.

### Effects of arylamides on mitochondrial function in MDA-MB-231 cells

The arylurea fatty acids from which arylamides 2b–6b were derived kill MDA-MB-231 cells by uncoupling OxPhos and inducing mitochondrial dysfunction. We therefore assessed the effects of the arylamides on mitochondrial function. We first measured oxygen consumption rates (OCR) in MDA-MB-231 cells treated with arylamides 2b–6b, as well as classical protonophores CCCP and DNP, using an XFe24 Seahorse analyser. Oxygen is consumed by the ETC during OxPhos, and OCR is expected to increase in cells treated with mitochondrial uncouplers as they increase respiration to compensate for increased proton leak.^12,39,40^ To carry out these assays, MDA-MB-231 breast cancer cells were treated with arylamides 2b–6b at the highest possible concentration of 40 µM, and OCR was monitored over 3 h. As shown in Fig. 2B, CCCP, DNP and all arylamides increased OCR and therefore stimulated respiration in MDA-MB-231 cells, but to differing extents. Thus 3,4-disubstituted aryl amides 5b and 6b produced the smaller increases in OCR than 3,5-disubstiuted aryl amides 2b–4b, and the classical protonophores CCCP (5 µM) and DNP (20 µM) had the greatest effect on OCR.

Next, the capacity of the arylamides, as well as CCCP and DNP, to depolarise the MIM in MDA-MB-231 cells was assessed using a JC-1 assay. JC-1 is a fluorescent cationic dye that accumulates in polarised mitochondria, forming aggregates that fluoresce red (≈590 nm). Upon depolarisation, JC-1 migrates to the cell cytosol where it disaggregates into monomers that fluoresce green (≈529 nm). Thus, the JC-1 red/green fluorescence intensity ratio can be used to determine the extent of mitochondrial depolarisation. In the JC-1 assays, MDA-MB-231 cells were treated with the arylamides over a range of concentrations for 1 h to capture early cellular effects of these compounds. JC-1 IC_50_ concentrations, defined as the concentration of test compounds required to shift the red/green fluorescence by 50% of control, and *E*_max_ values, defined as the maximum shift in JC-1 fluorescence ratio at the highest test concentration, were determined from dose-response curves (Fig. 2C and S1) and are shown in Table 1.

**Table 1:** JC-1 activity of arylamides 2b–6b, DNP and CCCP in MDA-MB-231 cells. Data represents the mean ± SEM of 3 independent experiments

Compound	Relative JC-1 IC_50_ (µM)	*E*_max_ (%)
2b	0.84 ± 0.37	15 ± 2
3b	4.0 ± 1.8	11 ± 1
4b	5.3 ± 1.7	12 ± 1
5b	5.5 ± 0.3	21 ± 1
6b	9.2 ± 1.4	34 ± 3
DNP	10.0 ± 1.7	14 ± 1
CCCP	4.0 ± 2.6	6 ± 1

All arylamides 2b–6b and CCCP and DNP were active in JC-1 assays, which indicates all compounds were able to diminish the proton gradients across the MIM in MDA-MB-231 cells. Combined with the increase in OCR observed in Seahorse data, these assays indicate that all test compounds are active mitochondrial uncouplers. The JC-1 IC_50_ concentrations of arylamides 2b–6b, DNP and CCCP, were all similar and fell within a concentration range of 0.81–9.99 µM, indicating that all compounds depolarise mitochondria with similar potencies.

Although all compounds affected the JC-1 ratio with similar potencies, the maximum level of depolarisation (*E*_max_) achieved by each compound varied. The *E*_max_ values for 3,5-disubstituted arylamides 2b–4b, as well as the full uncouplers DNP and CCCP, were all below 15%. In contrast, the *E*_max_ values of the 3,4-substituted arylamides 5b and 6b were 21.1 ± 1.2% and 34.1 ± 3.2%, respectively. The *E*_max_ values of 5b and 6b appear to reflect their maximum activity level, as their dose-response curves plateau at 20 µM with no significant change up to 40 µM. To provide further evidence that these *E*_max_ values were not an artefact of limited solubility in assay media we synthesised a short-chain (C12) analogue of 5b and assessed its JC-1 activity. This analogue could be assayed at concentrations up to 100 µM and showed a clear plateau from 40–100 µM with an *E*_max_ of 36.1 ± 4.2% (see Fig. S2). The results indicate that 3,5-disubstituted arylamides 2b, 3b and 4b, as well as DNP and CCCP, extensively depolarise mitochondria, while 5b and 6b cause only partial depolarisation, consistent with mild mitochondrial uncoupling.

To further characterise the uncoupling effect of the arylamides, we assessed their capacity to inhibit ATP production in MDA-MB-231 cells using the CellTiter-Glo 2.0 Assay. Full uncouplers such as DNP and CCCP inhibit ATP synthesis by collapsing mitochondrial proton gradients to the point where the PMF is no longer strong enough to drive proton flow through ATP-synthase,^9,41,42^ while mild uncoupling is expected to maintain the PMF at a level that supports ATP synthesis.

As shown in Fig. 2D, the 3,5-disubstituted arylamides (2b, 3b and 4b), DNP and CCCP, significantly decreased ATP production over 6 h, with the changes in ATP levels consistent with those produced by other full uncouplers.^43–45^ In contrast, ATP production was not significantly affected by 3,4-disubstituted arylamides 5b and 6b. To ensure that the observed changes in intracellular ATP levels were not caused by cell death, LDH release assays were performed on MDA-MB-231 cells treated for 6 h at 40 µM. LDH is an intracellular enzyme that is released as cells die and therefore serves as a marker of cell death. All test compounds failed to induce LDH release after 6 h (Fig. S3), meaning decreases in ATP are likely to result from uncoupling of OxPhos. Taken together, the *in vitro* data indicates that arylamides 5b and 6b weakly stimulate respiration, and partially depolarise mitochondria without affecting ATP production, and therefore act as mild mitochondrial uncouplers.

### Transmembrane proton transport rates of arylamides

We next sought to understand why arylamides 5b and 6b produce mild uncoupling, and to do this we considered the rates of proton flow across the MIM. In respiring mitochondria the ETC pumps protons across the MIM at a rate that maintains the proton gradient (ΔpH) and Δ*Ψ*_m_ a level high enough to support ATP-synthase activity.^46^ Mitochondrial ATP production is inhibited when ΔpH and Δ*Ψ*_m_ become too low, and ATP-synthase can instead catalyse the reverse reaction where ATP is hydrolysed. Introduction of a protonophore creates a new pathway for protons to flow in the opposite direction to the ETC and we hypothesised that the net effect – mild or full uncoupling – would be determined by the rate at which the protonophore transports protons across the MIM. High proton transport rates would result in extensive collapse the ΔpH and Δ*Ψ*_m_, leading to impaired ATP production and full uncoupling. In contrast, lower proton transport rates would lead to a partial reduction of ΔpH and Δ*Ψ*_m_ that still supports ATP synthesis. These are the hallmark characteristics of mild uncoupling. We therefore decided to investigate the transmembrane proton transport rates of the aryl amides in a vesicle-based assay.

For these studies, the HPTS vesicular assay was employed. This assay utilises vesicles containing specific salt species encapsulated within a lipid membrane, allowing proton transport activity to be isolated from other cellular processes present in more complex *in vitro* assays. This simplified system provides a direct analysis of the transport process. An aqueous solution (pH 7) of potassium gluconate (100 mM), HEPES buffer (10 mM), and HPTS (1 mM), a pH-sensitive fluorescent probe, was encapsulated within large unilamellar vesicles (200 nm) composed of 1-palmitoyl-2-oleoyl-*sn*-glycero-3-phosphocholine (POPC). These vesicles were suspended in a similar solution where HPTS was not present. A pH gradient was created across the vesicle membrane by adding a NaOH pulse to the external solution, mimicking the proton gradient found in the MIM.^47^ The compound being tested in this assay facilitates the dissipation of this gradient *via* proton transport, which is tracked by ratiometric changes in the emission wavelengths of the HPTS dye. After 200 s, the vesicles were lysed with detergent to release all encapsulated protons and provide a 100% efflux calibration.

Dose–response studies were conducted to generate Hill plots (Fig. S4–S13) and determine the EC_50_ value for each compound (Table 2), which represents the concentration of protonophore (mol%) required to achieve 50% proton efflux after 200 s and serves as a reliable indicator of protonophoric potency. The EC_50_ values for 2b–6b were similar and ranged from 0.041–0.14 mol%, which reflects their JC-1 IC_50_ concentrations. The Hill coefficients (*n*) provide insight into the relative stoichiometry of the transport event. Based on the proton transport mechanism shown in Fig. 1 Hill coefficients of 2 were expected as dimer formation is associated with the transport of a single proton. In all cases, Hill coefficients ranging from 1.4 to 1.8 were observed. These *n* values indicate the presence of both mono-deprotonated dimers and doubly deprotonated dimers, and have been observed for a related fatty acid protonophores in our previous work.^48^ Similar Hill coefficients across the series suggest that both classes of arylamides operate *via* the same mechanism.

**Table 2:** Summary of HPTS assay data, including EC_50_, Hill coefficients and initial rates of proton transport by arylamides 2b–6b (0.5 mol%)

Aryl amide	EC50^a^ (mol%)	*n* b	*k*_ib_ base first^c^^,^^d^ (% s^−1^)	*k*_ii_ 300 s incubation^c^^,^^e^ (% s^−1^)	*D* (*k*_ii_/*k*_ib_)^f^
2b	0.060 ± 0.003	1.8 ± 0.1	4.1	13	3.1
3b	0.041 ± 0.001	1.4 ± 0.1	3.7	18	4.9
4b	0.14 ± 0.001	1.8 ± 0.02	2.4	8.7	3.6
5b	0.08 ± 0.001	1.6 ± 0.01	2.6	6.8	2.7
6b	0.14 ± 0.009	1.7 ± 0.14	2.5	6.8	2.7

a EC_50_ concentrations reflect aryl amides concentrations with respect to the POPC concentration. HPTS assays were performed at POPC concentrations of 100 µM, thus EC_50_ concentrations have units of both mol% and µM.

b Hill coefficient, representing the transporter stoichiometry.

c Maximum initial rate calculated at a loading of 0.05 mol% by fitting the time-dependent % efflux trace to a single exponential, reported in “% efflux s^−1^” (the instantaneous rate at *t* = 0). Where an exponential was not appropriate, a linear fit to the initial segment was used, and the slope is reported in the same units.

d Rates obtained under a base-first protocol: base was first added to the external buffer, and the experiment was initiated by the subsequent addition of the transporter.

e Rates obtained under a 300 s incubation protocol: the transporter was added and incubated with vesicles for 300 s before the base pulse was added to initiate transport.

f Rate enhancement factor (*D* = *k*_ii_/*k*_ib_), quantifying the effect of 300 s incubation on initial rate.

Next, the rates at which the arylamides transport protons across the vesicle membranes at a loading of 0.5 mol% were determined. Rates were measured under two different assay conditions. In the first condition a NaOH base pulse was added to establish a proton gradient, and the rate (*k*_ib_) was measured immediately after addition of the test compound. In the second condition the aryl amide was added first and the experiment was initiated with a base pulse after a 300 s incubation period to give *k*_ii_. These assay conditions were used as previous studies have shown that anionophores insert into lipid bilayers at different rates, which affects their activity. The rates are shown in Table 2.

Without preincubation a clear distinction between the 3,5- and 3,4-disubstituted arylamides was not apparent. Although the 3,5-substituted compounds 2b and 3b exhibited the highest initial rates (*k*_ib_ = 4.1 and 3.7% s^−1^, respectively), the proton transport rate of the 3,5-dichloro arylamide 4b was similar to the 3,4-substituted analogues 5b and 6b. However the 300 s incubation provided a clear distinction in proton transport rated between the 3,5- and 3,4-disubstituted arylamides. Notably, compound 4b, which exhibited relatively modest proton transport rate without preincubation emerged as the third most active compound, achieving an initial rate of 8.7% s^−1^. Compounds 2b and 3b maintained the highest rates, with *k*_ii_ values of 13% s^−1^ and 18% s^−1^, respectively. In contrast, the 3,4-substituted analogues exhibited slower transport rates. Compound 5b remained the least active of the series, achieving an initial rate of 6.8% s^−1^ after 300 s of incubation.

The constant *D*, which represents the rate enhancement after 300 s incubation, was calculated to quantify the effects of incubation time on the initial proton transport rate. The values of *D* for the 3,5-substituted arylamides (2b–4b) are larger than those calculated for the 3,4-substituted compounds (5b and 6b), indicating that the 3,5-substituted arylamides require more time to insert into the lipid bilayer and benefit more from extended incubation. This trend highlights a key distinction between the two classes of arylamides. While the 3,4-substituted compounds appear to integrate more rapidly into the membrane, their overall rate of proton transport remains lower than that of their 3,5-substituted counterparts.

The results suggest that the aryl substitution pattern plays a crucial role in both the membrane insertion dynamics and the proton transport rates achieved by compounds 2b–6b. This role goes beyond lipophilicity, as the calculated log *P* values for the compounds vary minimally across the series (see Table S1). The 3,5-disubstituted compounds take longer to insert into the membrane, but once fully incorporated, transport protons at a markedly higher rates than their 3,4-disubstituted counterparts. These differences determine whether the compounds induce either mild or full mitochondrial uncoupling.

### Anion binding and dimerization of the arylamides

To identify possible causes of the different proton transport rates of the arylamides, the capacity of the compounds to self-assemble into membrane permeable anionic dimers was studied, as this forms part of the rate-limiting step in their protonophoric cycle.^37,49^ Dimer formation occurs *via* hydrogen bonding between an amide NH from one arylamide molecule with the carboxylate group of another. Therefore, the carboxylate (acetate) affinity of the amide groups in arylamides 2a–6a was assessed by ^1^H NMR titration experiments. The *tert*-butyl ester analogues of the arylamides (2a–6a) were titrated against *tert*-butyl ammonium acetate (TBAOAc) in acetone-*d*_6_. Solutions were titrated with up to 10 equiv. of TBAOAc, and changes in resonance attributed to the NH peak were tracked with increasing concentrations of TBAOAc (see Fig. S14–S18). The chemical shift of the arylamide NH shifted downfield initially before plateauing at higher equiv. The changes in chemical shift were inputted into the Bindfit applet and fitted to a 1 : 1 receptor:guest binding model.^50^ The binding constants of each arylamide *tert*-butyl ester are displayed in Table 3.

**Table 3:** Acetate binding constants (M^−1^), dimerisation constants (M^−1^), binding energies in water and benzene environments and dipole angles relative to the amide hydrogen bond axis in water and benzene environments of arylamides 2b–6b

Aryl amide	Binding constant^a^ (M^−1^)	Dimerisation constant^b^ (M^−1^)	Binding energy in water^c^ (kJ mol^−1^)	Binding energy in benzene^c^ (kJ mol^−1^)	Dipole angle in water^d^ (°)	Dipole angle in benzene^d^ (°)
2b	697 (±4%)	8275 ± 21	−87.1	−131.5	19.1	27.2
3b	937 ± 5	8484 ± 16	−95.6	−147.3	21.0	28.0
4b	1310 ± 1	9297 ± 18	−87.9	−128.6	16.7	23.4
5b	952 ± 4	2826 ± 24	−86.5	−143.3	27.9	35.7
6b	926 ± 10	2367 ± 13	−85.9	−131.4	22.4	30.5

a Acetate binding constants of arylamides in acetone-*d*_6_ at 298 K, calculated using a 1 : 1 binding model.^50^*Tert*-butyl arylamides 2a–6a were used for this study. Errors represent the uncertainties of the fitted binding constant (%). See SI for dissociation constants in µM.

b Dimerisation constants of 2b–6b calculated from the aromatic C–H peak chemical shifts of deprotonated 2b–6b at 5 µM–5 mM in CDCl_3_ when fitted into a monomer-dimer aggregation model.^50,51^ Errors represent the uncertainties of the fitted dimerisation constant (%). See SI for dissociation constants in µM.

c Binding energies of dimers formed by a protonated and deprotonated arylamide were evaluated at the M06-2X-D3/6-31G(d,p)//M06-2X-D3/6-311++G(2df,2p) level of theory in water and benzene environments.

d Dipole angles of arylamides 2b–6b calculated at the M06-2X-D3/6-31G(d,p)//M06-2X-D3/6-311++G(2df,2p) level of theory in water and benzene environments.^52^ Dipole angles for 2b, 5b and 6b were calculated by taking into account the dipole angles of each conformer and their Boltzmann distributions.

All arylamides bound to acetate with moderate affinity, and there was no clear distinction between the 3,5- and 3,4-substituted arylamides. Indeed, the full uncoupler 4a had the lowest acetate affinity in the series and the mild uncoupler 6a had the highest acetate affinity.

We next assessed the capacity of the arylamides to dimerise using concentration-dependent ^1^H NMR titration studies in CDCl_3_. Deprotonation of the molecules was induced using 1 equiv. of tetrabutylammonium hydroxide (TBAOH). Increasing concentrations of (2b–6b)-TBAOH from 5 µM–5 mM caused downfield shifts in the resonances attributed to the aromatic C–H protons (see Fig. S19–S23). The concentration-dependant shifts in these resonances were fitted into a monomer-dimer aggregation model to give dimerization constants for 2b–6b (Table 3).^50^

In contrast to the acetate binding constants, a clear difference was observed between the dimerization constants of the 3,4- and 3,5-disubstituted arylamides. Dimerization constants for the 3,5-disubstituted arylamides 2b–4b were all above 8200 M^−1^, while those of the 3,4-substituted arylamides 5b and 6b were 2826 ± 24 and 2367 ± 12, respectively. Compound 4b with a 3,5-dichloro head group had the greatest dimerisation constant of 9297 M^−1^ whilst its 3,4-substituted counterpart 6b had the weakest dimerisation constant with 2367 M^−1^. These data suggest that the superior protonophoric activities of arylamides 2b–4b arises because they form head-to-tail dimers more readily than 5b and 6b.

Interestingly, the dimerization constants of 2b–6b do not correlate with the electron withdrawing capacity of the aromatic substituents (as determined from their Hammett's substituent constants, see Table S1). For example, 5b has the strongest electron withdrawing substitution pattern in the series but has one of the lowest dimerization constants. This is unexpected as the hydrogen bonding donating strength of anionophores is enhanced by electron withdrawing groups.^37,53,54^ A computational evaluation was therefore performed.

### Computational evaluation of arylamide dimers

Initially the energetics of dimer formation was evaluated. Binding energies of dimers formed by a protonated and deprotonated arylamide were evaluated at the M06-2X-D3/6-31G(d,p)//M06-2X-D3/6-311++G(2df,2p) level of theory and are shown in Table 3. Complexes were examined in both water (to mimic the intermembrane space/matrix) and benzene (to mimic the membrane environment) using implicit solvation. In the hydrophilic environment, dimer formation of 3,5-substituted arylamides 2b–4b was more energetically favourable in comparison to 3,4-substituted arylamides 5b and 6b (Table 3). However, in the hydrophobic environment this trend is not as apparent, as some 3,4-substituted arylamides formed more stable dimers than 3,5-substituted arylamides, such as 5b with the second greatest binding energy of −143.3 kJ mol^−1^.

In the protonophoric cycle 2b–6b dimerise at the water-MIM interface, which is hydrophilic. The differences in trends between the modelled membrane (benzene) and solution (water) environments infer that dimer formation at the membrane interface is an important determinant of protonophoric activity, rather than the rate that which these dimers traverse the hydrophobic core of the MIM. Specifically, 3,5-disubstituted arylamides can form more stable dimers before moving across the MIM, which leads to greater proton transport rates, as a result causing full mitochondrial uncoupling. In contrast, 3,4-disubstituted arylamides form less stable dimers which causes proton transport rates to decrease, thus leading to mild uncoupling.

The superior dimerization of the 3,5-disubstituted arylamides indicates a role for substitution pattern, which we reasoned could arise from differences in distribution and orientation of electron density around the aromatic ring of arylamides. Notably, changes in the electron density can affect the NH group's ability to act as a hydrogen bond donor when forming a dimeric complex with a carboxylate. Measuring the dipole angle of the aromatic ring relative to the amide hydrogen bond axis provides a measure of the partial positive charge of the NH moiety, where a better alignment indicates a greater partial positive charge on the NH group and therefore a stronger hydrogen bonding ability.^55–57^

**Fig. 3:**
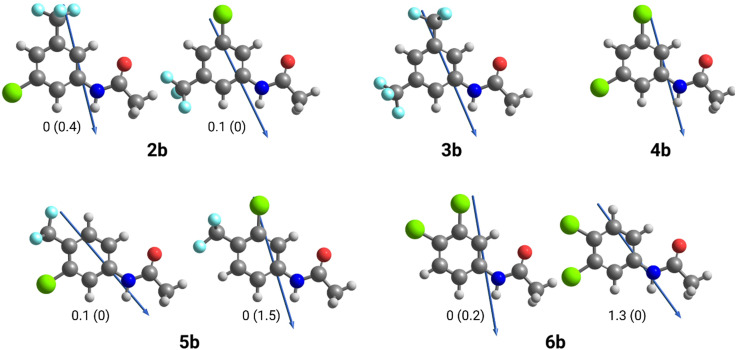
Depiction of dipole angles relative to amide hydrogen bond axis of arylamides. Due to their unsymmetrical substitution, two different conformations of 2a, 5b and 6b were considered. Relative energies in kJ mol^−1^ are shown for each conformation. Values in brackets represent solvation in benzene, while values outside of brackets represent solvation in water. Dipoles point from negative to positive.

To investigate this effect, the dipole angles of the aryl substituents relative to the amide hydrogen bond axis of 2b–6b were calculated at the M06-2X-D3/6-31G(d,p)//M06-2X-D3/6-311++G(2df,2p) level of theory (Table 3). Due to the unsymmetrical substitution patterns in arylamides 2b, 5b and 6b, the dipole angle will differ depending on their conformation (Fig. 3). As the calculated energy differences between conformers are low (<2 kJ mol^−1^, see Fig. 3), the two conformations will exist in equilibrium with one another. To account for this equilibrium, average dipole angles for each set of conformers were calculated by considering the probabilities of each conformer. Probabilities were calculated using the energies in each environment and applying them to a Boltzmann Distribution (see SI for calculations).

As shown in Table 3, the 3,5-disubstituted arylamides 2b–4b had smaller dipole angles relative to the amide hydrogen bond axis than 3,4-disubstituted arylamides 5b and 6b in benzene and water environments. In both environments, mild uncoupler 5b had the highest dipole angle relative to the amide hydrogen bond axis whilst full uncoupler 4b had the lowest dipole angle. The smaller dipole angles infer that the dipole of the 3,5-disubstituted aromatic ring aligns better within the polarisation of the arylamide NH group, therefore promoting dimerization *via* hydrogen bonding. In contrast, the 3,4-disubstituted arylamides had greater dipole angles in both environments, inferring that the substituent's dipole does not align as well with the polarisation of the amide group and accounts for less efficient dimer formation. Indeed, the trends in dipole alignment across the series align with the experimentally determined dimerisation constants (Table 3), where 3,5-disubstituted arylamides 2b–4b had greater dimerisation affinities than 3,4-disubstituted arylamides 5b and 6b.

This computational evaluation provides justification as to why 3,4-disubstituted arylamides form less stable dimeric complexes in comparison to 3,5-disubstituted arylamides and given the importance of dimerization in the protonophoric cycle, why the 3,4-disubstituted arylamides possess diminished proton transport activity. The evaluation also suggests that substitution pattern can influence anionophore activity and should be considered in the design of new anion receptors.

## Conclusions

In this paper we present the first examples of protonophores that act exclusively as mild uncouplers. 3,4-Disubstitution of arylamide substituted fatty acids, an atypical anionophore-based scaffold, produced protonophores that weakly stimulated respiration and partially depolarised mitochondria without affecting ATP production. These effects are hallmarks of mild uncoupling, and occurred regardless of concentration. Mechanistic studies revealed the diminished capacity of these mild uncouplers to form membrane permeable dimers in the rate limiting step of their protonophoric cycles, which resulted in a slower rates of transmembrane proton transport rates. By demonstrating a link between mild uncoupling and proton transport rate, we anticipate that these findings will provide a new conceptual framework to explore mild uncoupler development and accelerate the discovery of safe and effective therapeutic uncoupling agents.

## Author contributions

Conceptualization: T. R., P. A. G and D. A. M.; investigation: E. P., D. A. M., K. A. W. and A. P.; formal analysis: E. P, D. A. M., K. A. W. and A. P.; methodology: T. R., P. A. G., D. A. M. and K. A. W.; supervision: T. R., P. A. G. and K. A. W.; writing – original draft E. P and D. A. M.; writing – review and editing: all authors.

## Conflicts of interest

The authors report no conflicts of interest.

## Supplementary Material

SC-OLF-D5SC06530E-s001

## Data Availability

The data supporting this article have been included as part of the supplementary information (SI). Supplementary information is available. See DOI: https://doi.org/10.1039/d5sc06530e.
