# Supplementary material for: The role of transmembrane proton transport rates in mild mitochondrial uncoupling by arylamide substituted fatty acids
Source: Chem Sci. 2025 Dec 2;17(7):3506–15. doi: 10.1039/d5sc06530e (PMC12728566; doi:10.1039/d5sc06530e)
Supplement: SC-017-D5SC06530E-s001 [file SC-017-D5SC06530E-s001.pdf]

Supplementary Information (SI) for Chemical Science.
This journal is © The Royal Society of Chemistry 2025

Supplementary Information

The role of transmembrane proton transport rate in mild mitochondrial uncoupling by arylamide substituted fatty acids.

Ethan Pacchini,[1] Daniel Mcnaughton,[1] Aaron Pye,[2] Katie Wilson,[2] Philip A. Gale,[1] Tristan Rawling[1]

[1]School of Mathematical and Physical Sciences, Faculty of Science, University of Technology Sydney, Sydney, NSW, 2007, Australia

[2]Department of Biochemistry, Memorial University of Newfoundland, St John's, NL A1C 5S7, Canada

Table of Contents

1. Supporting Tables

2. Supporting Figures – Cell Culture Assays

3. Supporting Figures – HPTS Assay

4. Supporting Figures – ^{1}H NMR TBAOAc Titration Stack Plots

5. Supporting Figures – ^{1}H NMR TBAOH Titration Stack Plots

6. Supporting Figures – Computational Evaluation

7. Experimental Procedures – Chemistry

8. Experimental Procedures – Cell Culture

9. Experimental Procedures – HPTS Assay

10. Experimental Procedures – NMR Studies

10. Experimental Procedures – Computational Evaluation

11. NMR Characterisation

12. References

1. Supporting Tables

Table S1: Calculated hammett substitution values and cLogP of aryl amides **2b-6b.**

Aryl Amide	σ_{total}[a]	cLogP[b]
**2b**	0.80	7.27
**3b**	0.86	7.19
**4b**	0.74	6.94
**5b**	0.91	7.27
**6b**	0.60	6.94

[a] Hammett substituent constants were taken from published values[1]

[b] cLogP values were calculated using ALOGP 2.1[2]

Table S2: Acetate and dimer dissociation constants of aryl amides **2b-6b**

Aryl Amide	Acetate dissociation constant (µM)[a]	Dimer dissociation Constant (µM)[b]
**2b**	1430	121
**3b**	1070	118
**4b**	763	108
**5b**	1050	354
**6b**	1080	422

[a] Calculated from acetate binding constants presented in Table 3
[b] Calculated from dimerization constants presented in Table 3

2. Supporting Figures – Cell Culture Assays

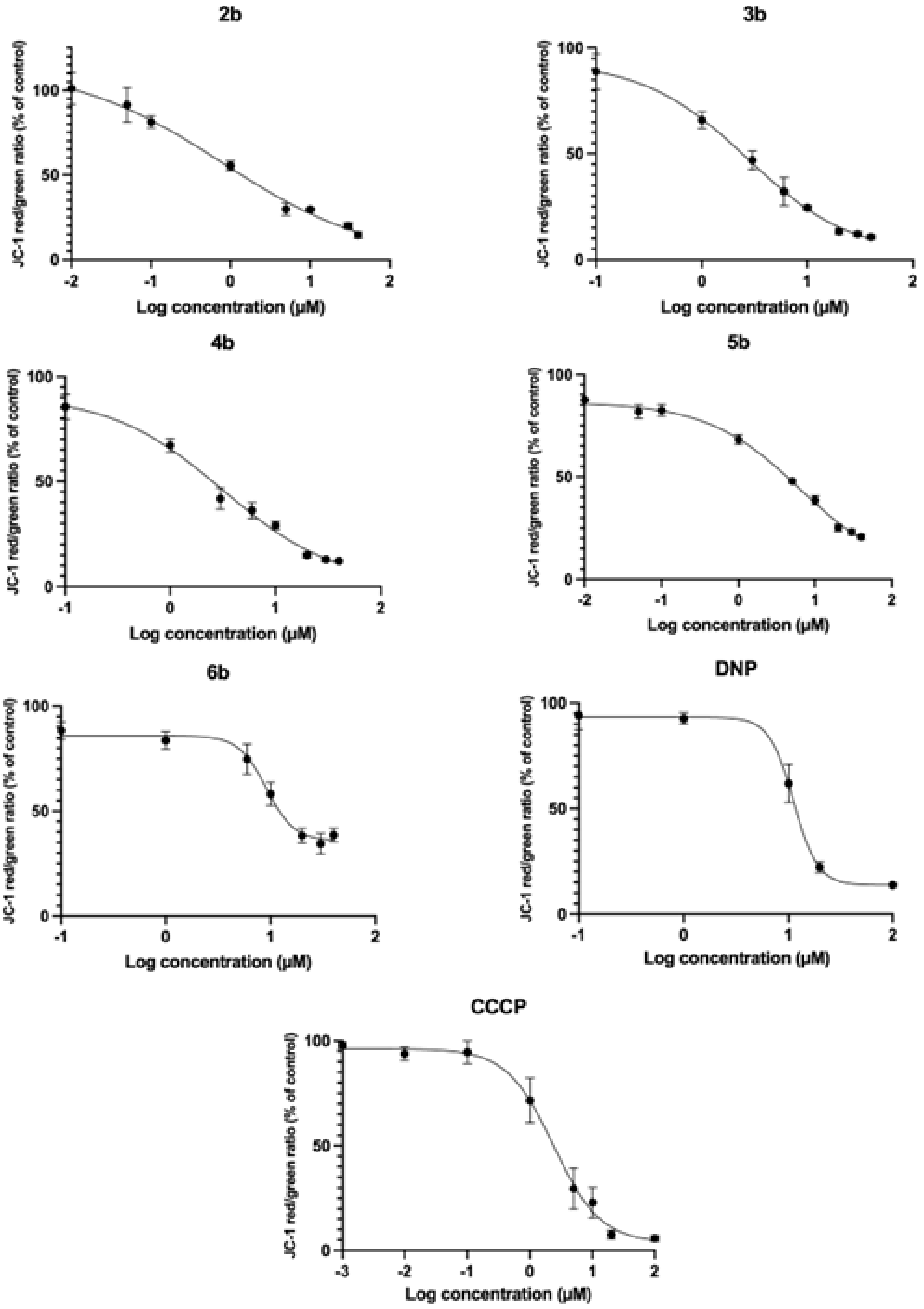

Figure S1: Dose-response curves showing the effects of arylamides **5b-6b**, DNP and CCCP (1 h) on the JC-1 red:green fluorescence ratio in MDA-MB-231 breast cancer cells relative to DMSO control. Data represents mean ± SEM of three independent experiments.

(a)

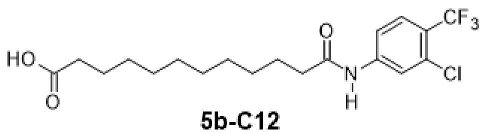

5b-C12

(b)

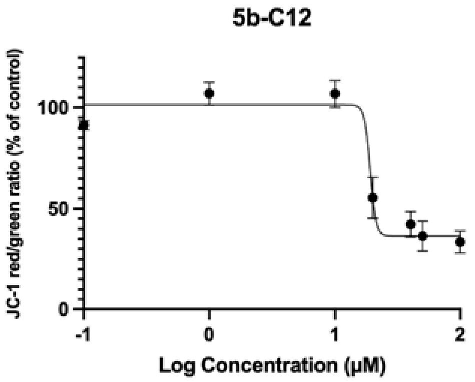

JC-1 IC_{50} = 22.4 ± 3.6 μM

E_{max} = 36.1 ± 4.2 %.

Figure S2: Chain-shortened analogue of arylamide 5b. **(a)** Chemical structure of **5b-C12**; **(b)** Dose-response curve showing the effect of **5b-C12** (1 h) on the JC-1 red:green fluorescence ratio in MDA-MB-231 breast cancer cells relative to DMSO control. Data represents mean ± SEM of three independent experiments.

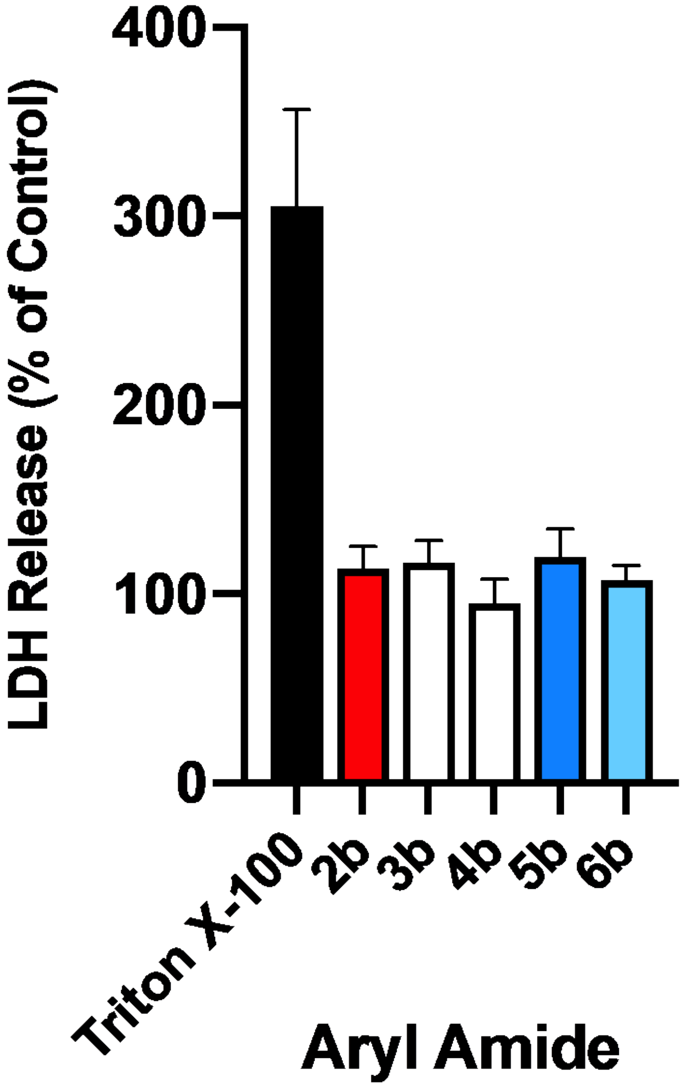

Figure S3: **Effects of arylamides on LDH release**: LDH release of MDA-MB-231 cells when treated with aryl amides **2b-6b** at 40 µM for 6 h relative to DMSO control. Triton X-100 was used as a positive control which exhibits maximal LDH release.

3. Supporting Figures – HPTS Assay

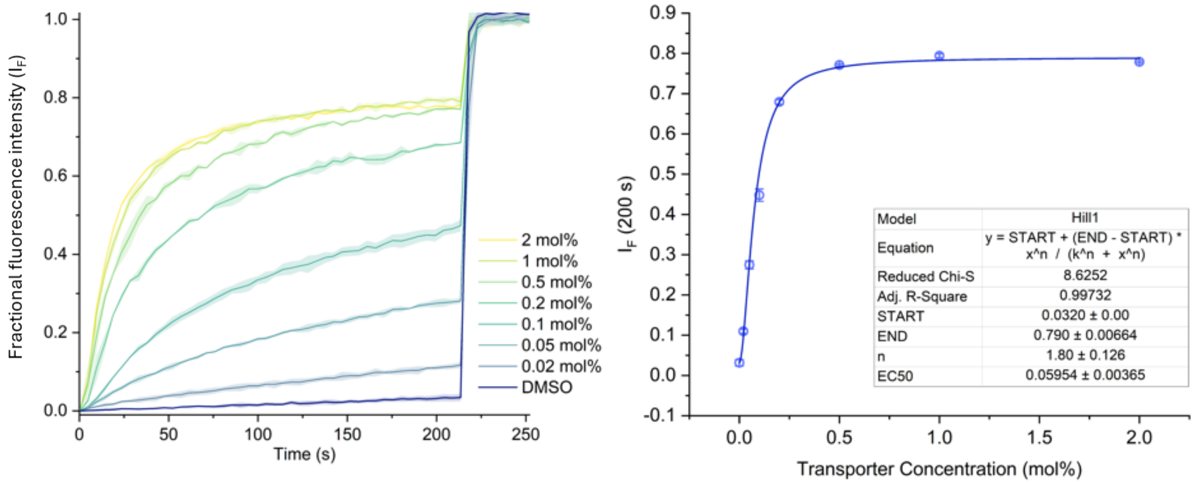

Figure S4: Hill plot analysis of H^+/OH^- transport facilitated by compound **2b** measured using the KGluc assay. NaOH (5 mM) and valinomycin (0.05 mol%) were added to the vesicles before the addition of **2b** at 0 s. Detergent was added at $t = 210$ s to lyse the vesicles. Compound concentrations are shown as compound-to-lipid molar ratios. Error bars represent standard deviations from two repeats.

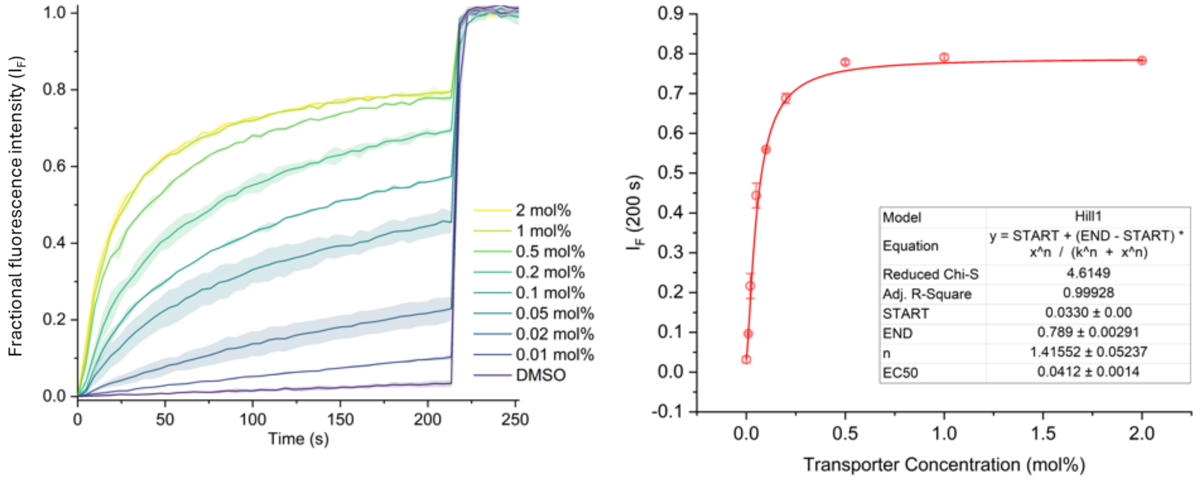

Figure S5: Hill plot analysis of H^+/OH^- transport facilitated by compound **3b** measured using the KGluc assay. NaOH (5 mM) and valinomycin (0.05 mol%) were added to the vesicles before the addition of **3b** at 0 s. Detergent was added at $t = 210$ s to lyse the vesicles. Compound concentrations are shown as compound-to-lipid molar ratios. Error bars represent standard deviations from two repeats.

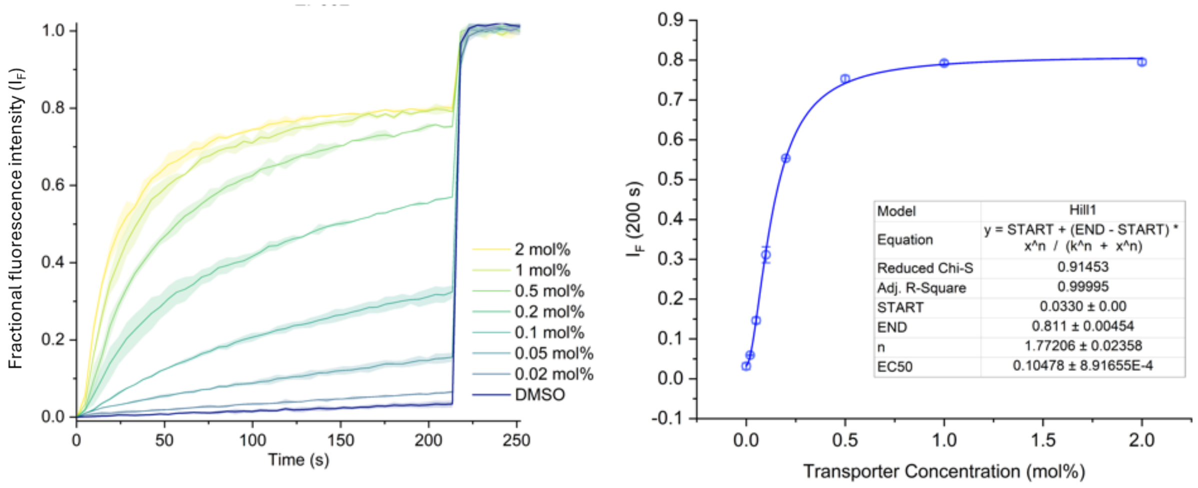

Figure S6: Hill plot analysis of H^+/OH^- transport facilitated by compound **4b** measured using the KGluc assay. NaOH (5 mM) and valinomycin (0.05 mol%) were added to the vesicles before the addition of **4b** at 0 s. Detergent was added at $t = 210$ s to lyse the vesicles. Compound concentrations are shown as compound-to-lipid molar ratios. Error bars represent standard deviations from two repeats.

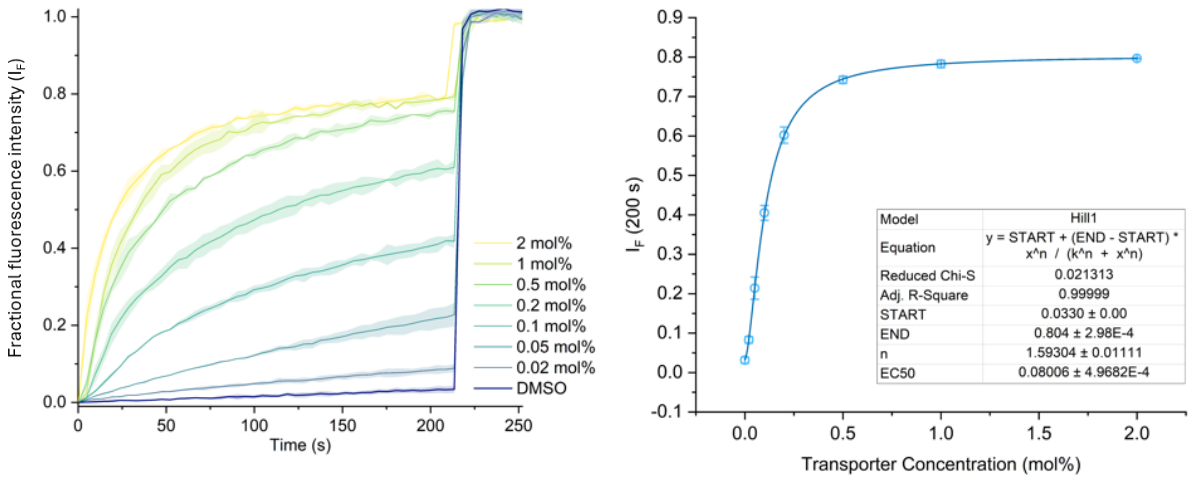

Figure S7: Hill plot analysis of H^+/OH^- transport facilitated by compound **5b** measured using the KGluc assay. NaOH (5 mM) and valinomycin (0.05 mol%) were added to the vesicles before the addition of **5b** at 0 s. Detergent was added at $t = 210$ s to lyse the vesicles. Compound concentrations are shown as compound-to-lipid molar ratios. Error bars represent standard deviations from two repeats.

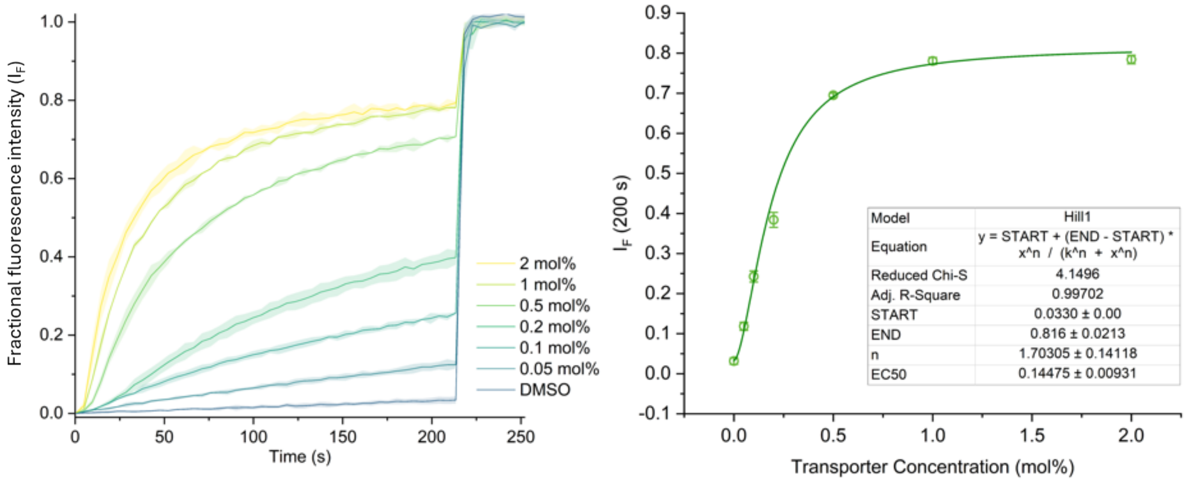

Figure S8: Hill plot analysis of H^+/OH^- transport facilitated by compound **6b** measured using the KGluc assay. NaOH (5 mM) and valinomycin (0.05 mol%) were added to the vesicles before the addition of **6b** at 0 s. Detergent was added at $t = 210$ s to lyse the vesicles. Compound concentrations are shown as compound-to-lipid molar ratios. Error bars represent standard deviations from two repeats.

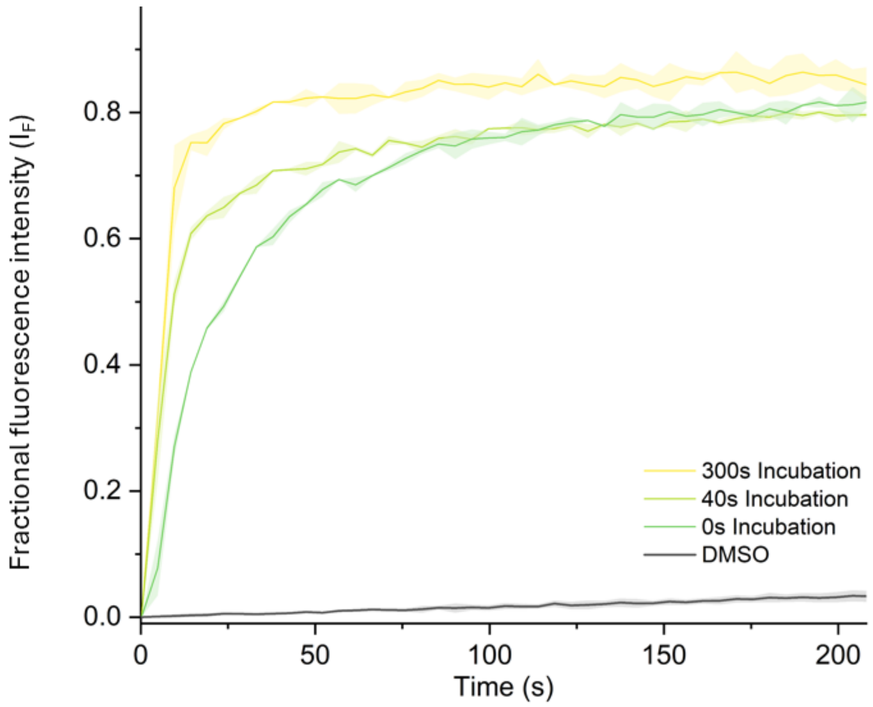

Figure S9: The HPTS efflux plots for compound **2b** (0.5 mol%) measured using the KGluc assay under three incubation conditions. Valinomycin (0.05 mol%) were added to the vesicles in each experiment before the addition of **2b** and NaOH (5 mM) in various orders. 0 s incubation represents base-first addition followed by protonophore initiation. 40 s incubation represents protonophore-first addition followed by base initiation. 300 s incubation represents protonophore first addition followed by base initiation after a 300 s period. Detergent was added at $t = 210$ s to lyse the vesicles. Error bars represent standard deviations from two repeats.

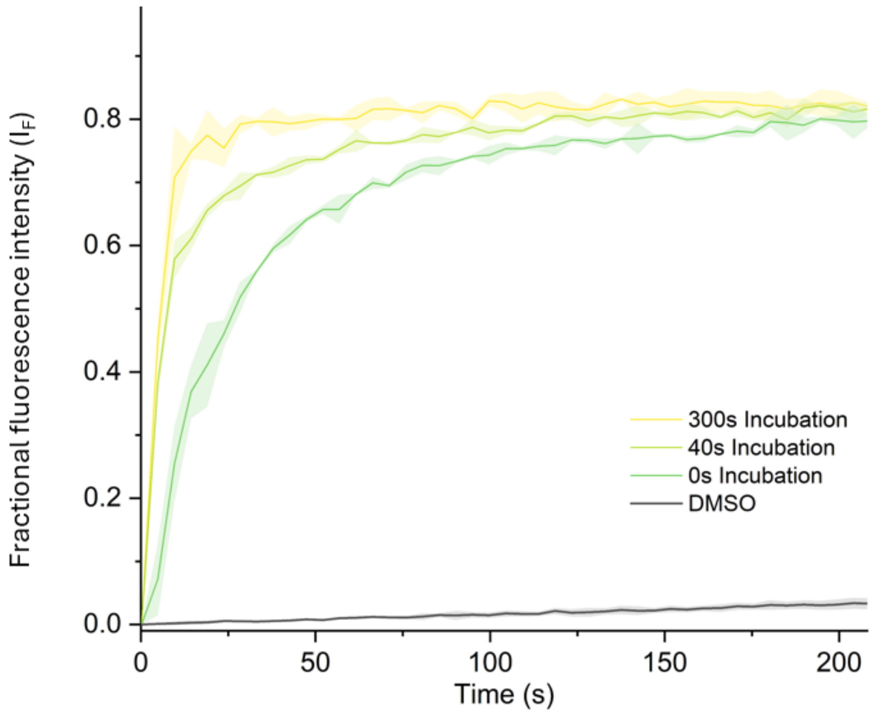

Figure S10: The HPTS efflux plots for compound **3b** (0.5 mol%) measured using the KGluc assay under three incubation conditions. Valinomycin (0.05 mol%) were added to the vesicles in each experiment before the addition of **3b** and NaOH (5 mM) in various orders. 0 s incubation represents base-first addition followed by protonophore initiation. 40 s incubation represents protonophore-first addition followed by base initiation. 300 s incubation represents protonophore first addition followed by base initiation after a 300 s period. Detergent was added at $t = 210$ s to lyse the vesicles. Error bars represent standard deviations from two repeats.

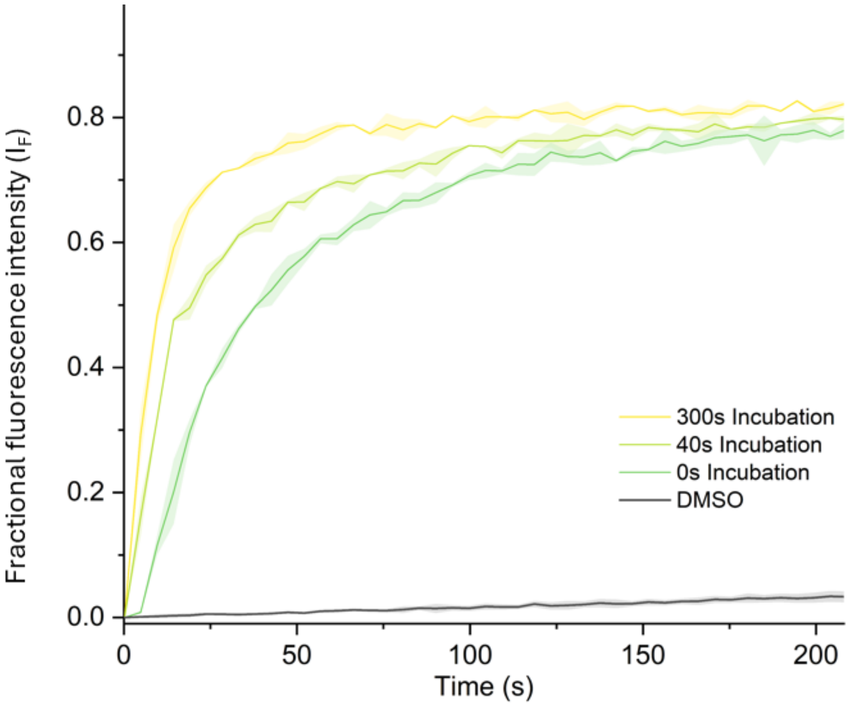

Figure S11: The HPTS efflux plots for compound **4b** (0.5 mol%) measured using the KGluc assay under three incubation conditions. Valinomycin (0.05 mol%) were added to the vesicles in each experiment before the addition of **4b** and NaOH (5 mM) in various orders. 0 s incubation represents base-first addition followed by protonophore initiation. 40 s incubation represents protonophore-first addition followed by base initiation. 300 s incubation represents protonophore first addition followed by base initiation after a 300 s period. Detergent was added at $t = 210$ s to lyse the vesicles. Error bars represent standard deviations from two repeats.

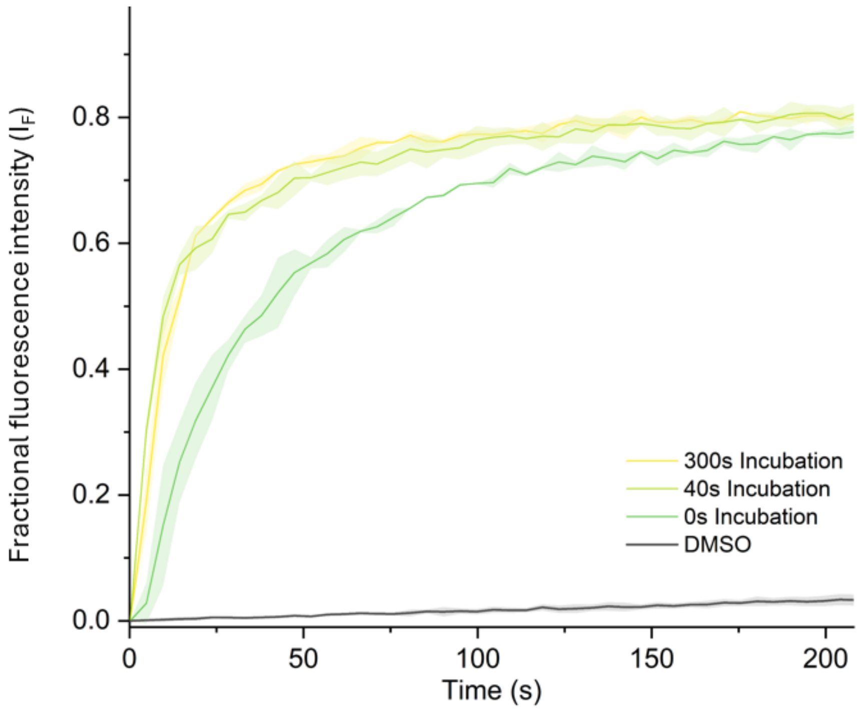

Figure S12: The HPTS efflux plots for compound **5b** (0.5 mol%) measured using the KGluc assay under three incubation conditions. Valinomycin (0.05 mol%) were added to the vesicles in each experiment before the addition of **5b** and NaOH (5 mM) in various orders. 0 s incubation represents base-first addition followed by protonophore initiation. 40 s incubation represents protonophore-first addition followed by base initiation. 300 s incubation represents protonophore first addition followed by base initiation after a 300 s period. Detergent was added at $t = 210$ s to lyse the vesicles. Error bars represent standard deviations from two repeats.

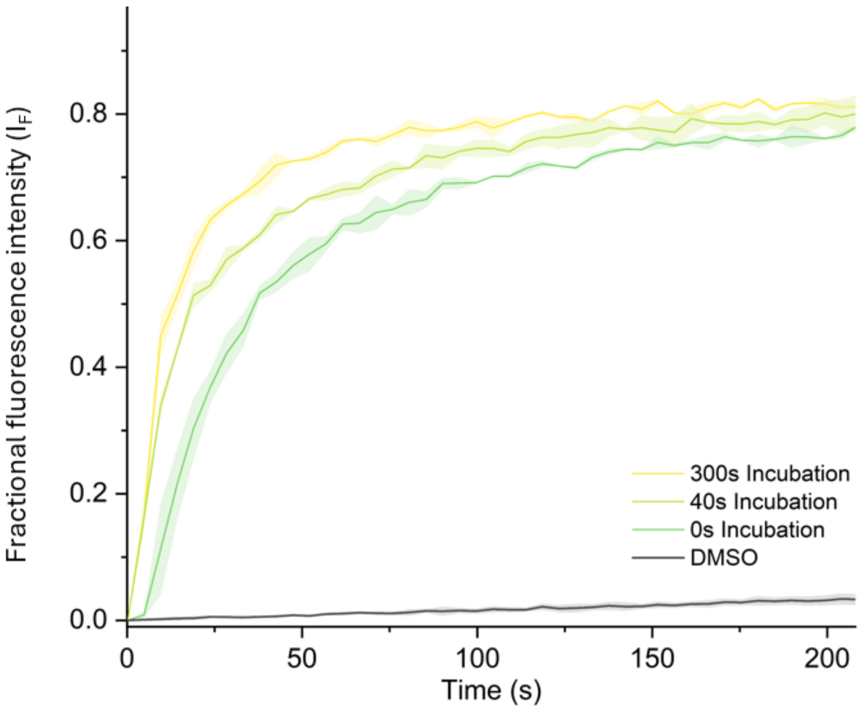

Figure S13: The HPTS efflux plots for compound **6b** (0.5 mol%) measured using the KGluc assay under three incubation conditions. Valinomycin (0.05 mol%) were added to the vesicles in each experiment before the addition of **6b** and NaOH (5 mM) in various orders. 0 s incubation represents base-first addition followed by protonophore initiation. 40 s incubation represents protonophore-first addition followed by base initiation. 300 s incubation represents protonophore first addition followed by base initiation after a 300 s period. Detergent was added at $t = 210$ s to lyse the vesicles. Error bars represent standard deviations from two repeats.

4. Supporting Figures – ^{1}H NMR TBAOAc Titration Stack Plots

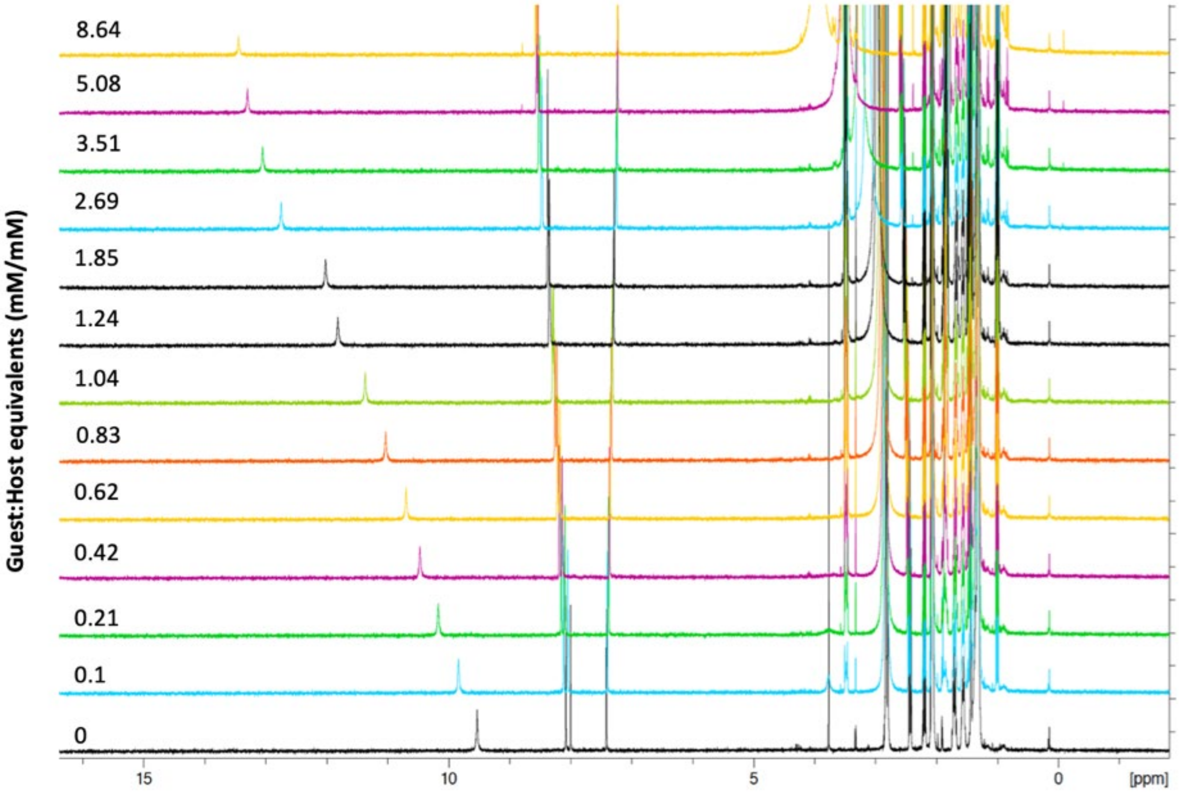

Figure S14: ^{1}H NMR titration spectra as a stack plot for aryl amide ester **2a** + TBAOAc in acetone-d_6 at 298 K. OAc^{-} binding constant determined was 696.58 M^{-1} calculated by fitting the change in aryl amide N-H chemical shift to a 1:1 binding model on bindfit with changing [OAc^{-}] from 0-8 mM/mM equivalents guest/host. Full plot details can be found using the following link: http://app.supramolecular.org/bindfit/view/1514c8b3-3e72-419f-a53d-b7484aa4abab

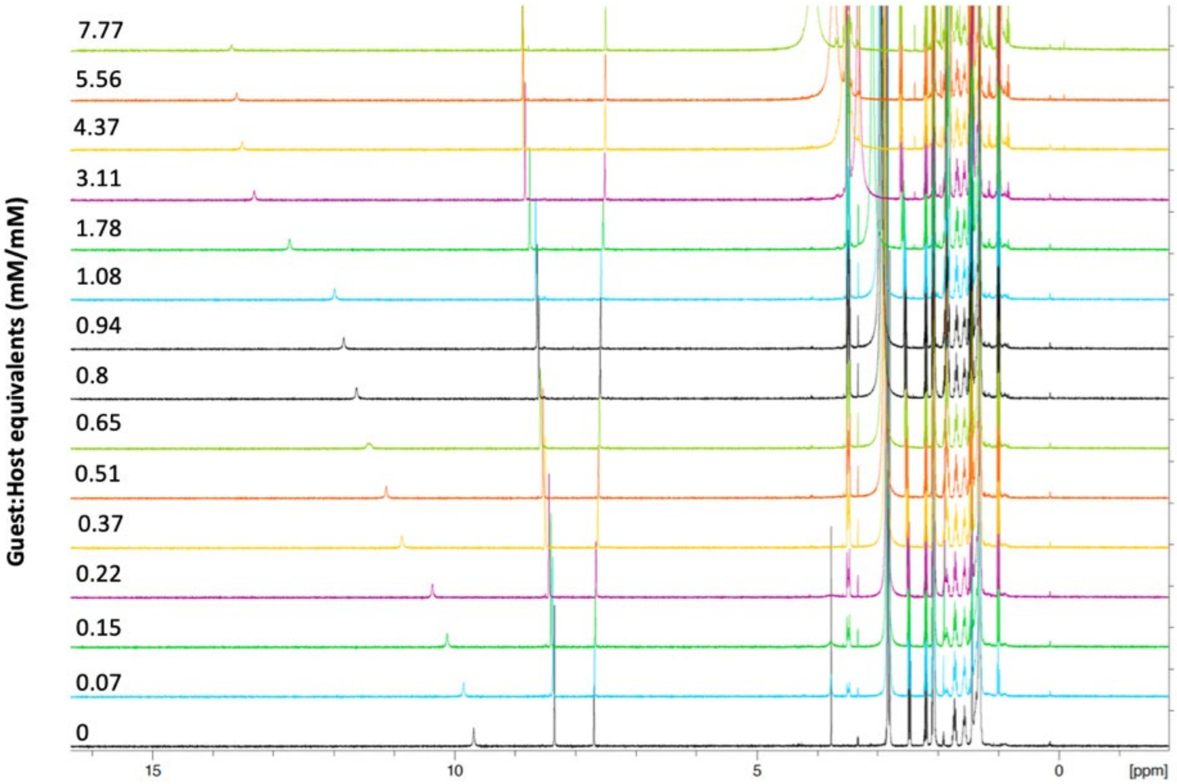

Figure S15: ^{1}H NMR titration spectra as a stack plot for aryl amide ester **3a** + TBAOAc in acetone-d_6 at 298 K. OAc^{-} binding constant determined was 936.59 M^{-1} calculated by fitting the change in aryl amide N-H chemical shift to a 1:1 binding model on bindfit with changing [OAc^{-}] from 0-8 mM/mM equivalents guest/host. Full plot details can be found using the following link: http://app.supramolecular.org/bindfit/view/b82b2376-ca35-417a-bd6e-591a2e05b24e

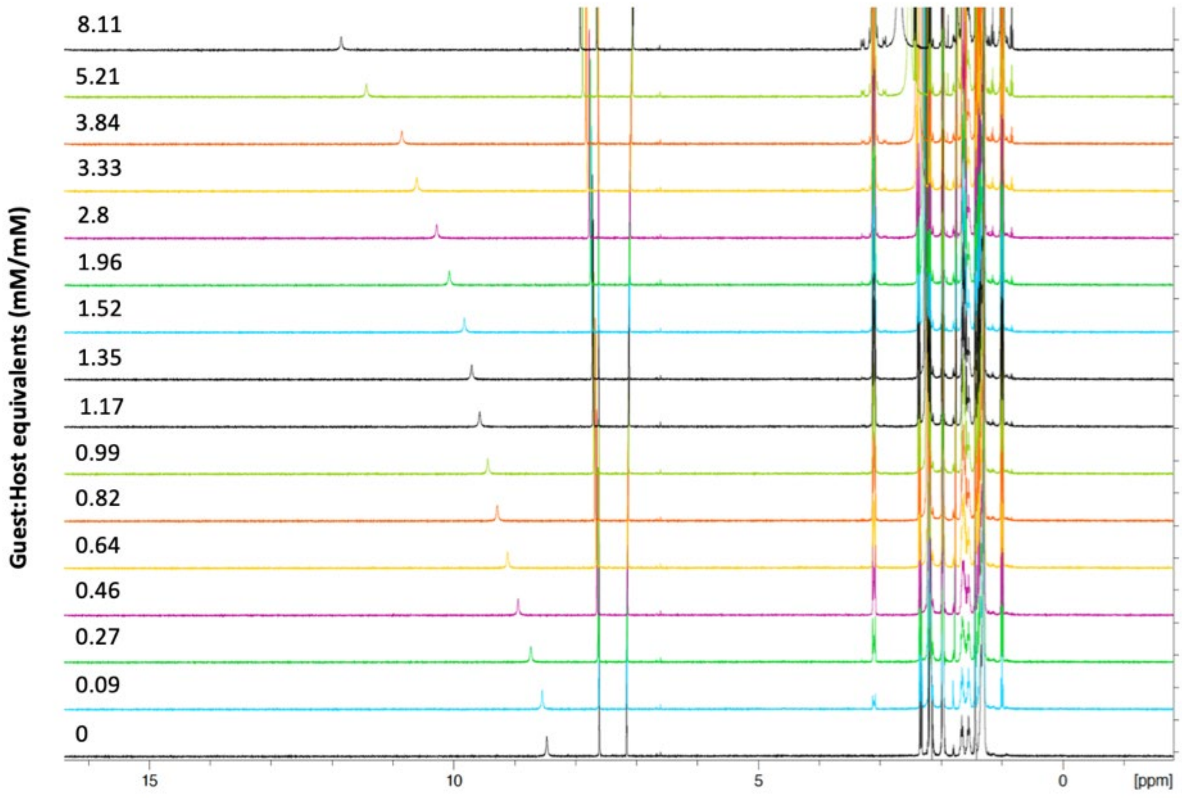

Figure S16: ^{1}H NMR titration spectra as a stack plot for aryl amide ester **4a** + TBAOAc in acetone-d_6 at 298 K. OAc^{-} binding constant determined was 1306.84 M^{-1} calculated by fitting the change in aryl amide N-H chemical shift to a 1:1 binding model on bindfit with changing [OAc^{-}] from 0-8 mM/mM equivalents guest/host. Full plot details can be found using the following link: http://app.supramolecular.org/bindfit/view/f4dd8115-47ca-4123-b567-0cc5c188e8ba

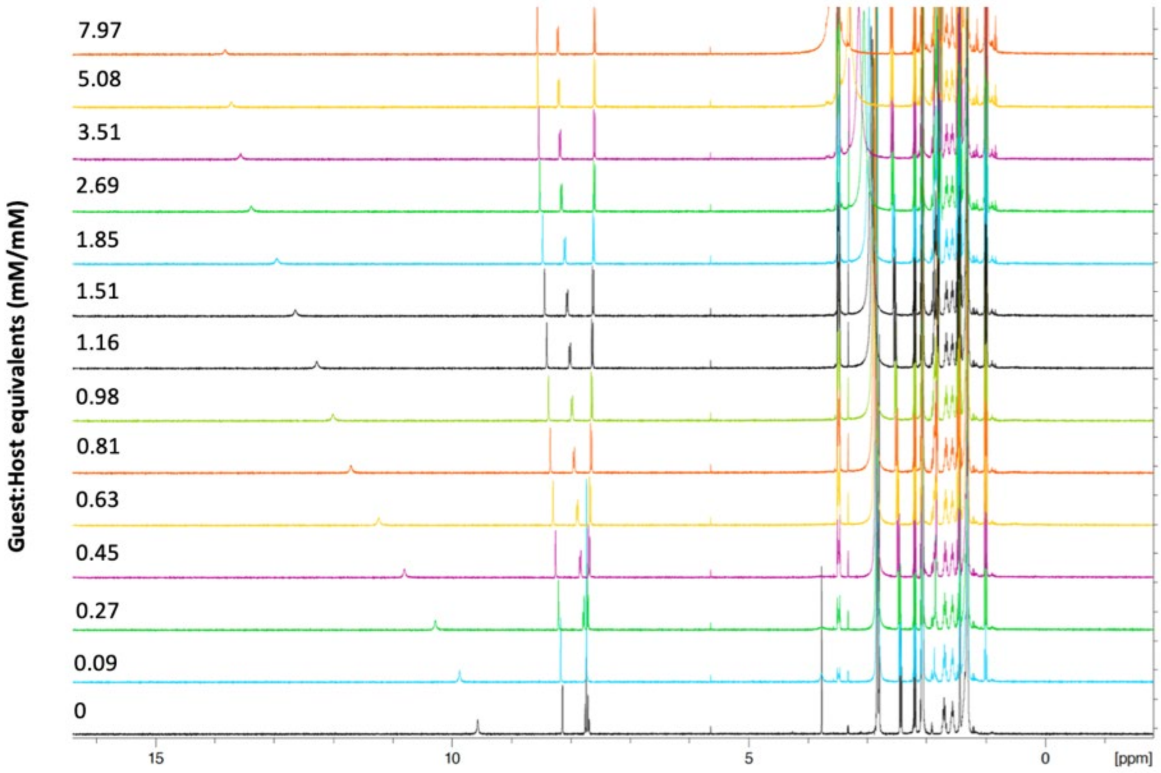

Figure S17: ^{1}H NMR titration spectra as a stack plot for aryl amide ester **5a** + TBAOAc in acetone-d_6 at 298 K. OAc^{-} binding constant determined was 818.91 M^{-1} calculated by fitting the change in aryl amide N-H chemical shift to a 1:1 binding model on bindfit with changing [OAc^{-}] from 0-8 mM/mM equivalents guest/host. Full plot details can be found using the following link: http://app.supramolecular.org/bindfit/view/fe0176a1-9258-4168-96f6-a26c6b76e236

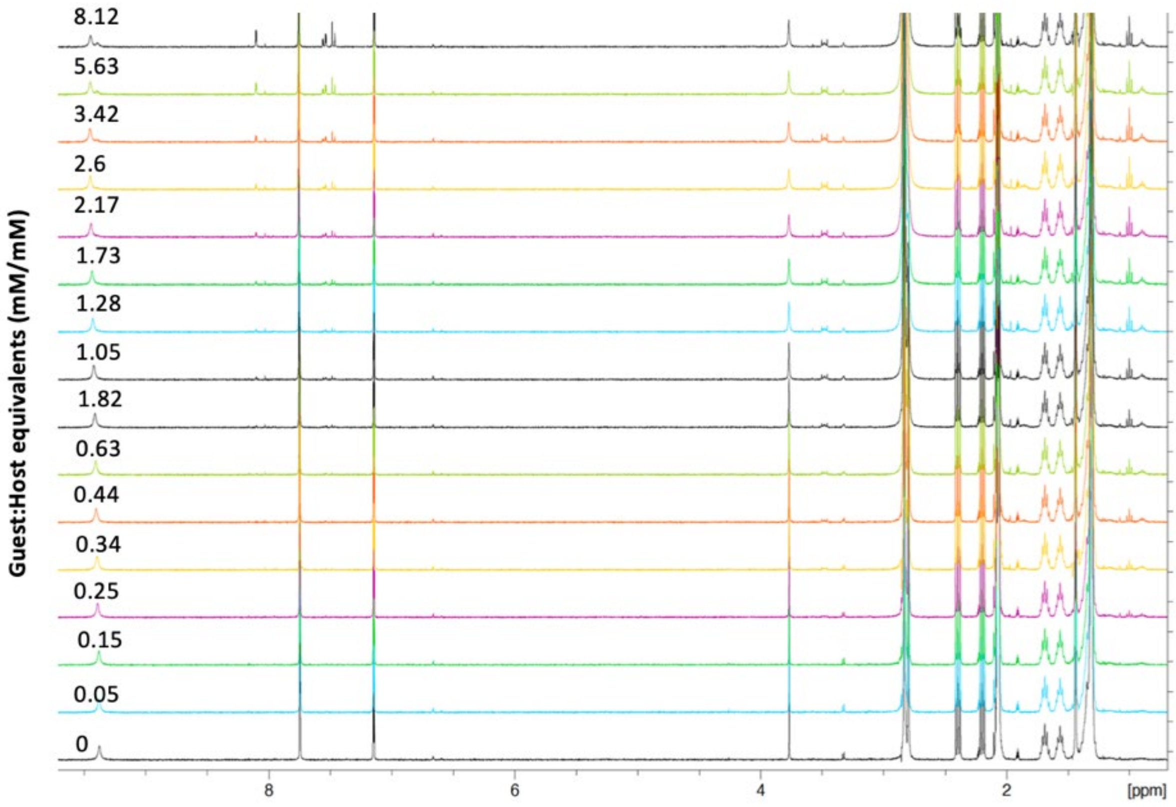

Figure S18: ^{1}H NMR titration spectra as a stack plot for aryl amide ester **6a** + TBAOAc in acetone-d_6 at 298 K. OAc^{-} binding constant determined was 936.10 M^{-1} calculated by fitting the change in aryl amide N-H chemical shift to a 1:1 binding model on bindfit with changing [OAc^{-}] from 0-8 mM/mM equivalents guest/host. Full plot details can be found using the following link: http://app.supramolecular.org/bindfit/view/22a84dca-1990-4be8-9bf7-0391e2a25b5a

5. Supporting Figures – ^{1}H NMR TBAOH Titration Stack Plots

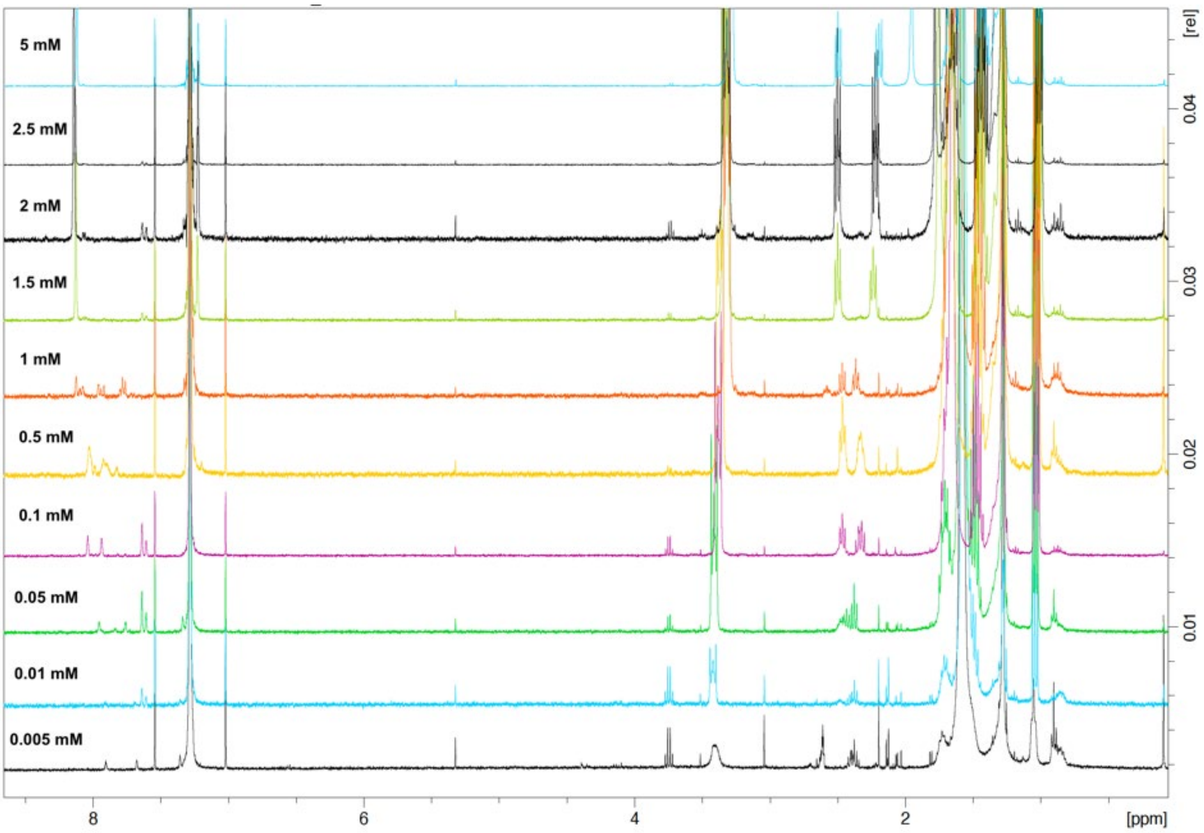

Figure S19: Concentration-dependent ^{1}H NMR titration spectra as a stack plot for aryl amide **2b** + TBAOH in $CDCl_3$ at 298 K. Dimerisation constant determined was 8274.73 M^{-1} and calculated by fitting the average chemical shifts of two aromatic C-H peaks to a NMR Dimer Aggregation model on bindfit with concentrations of **2b** between 5-0.005 mM.

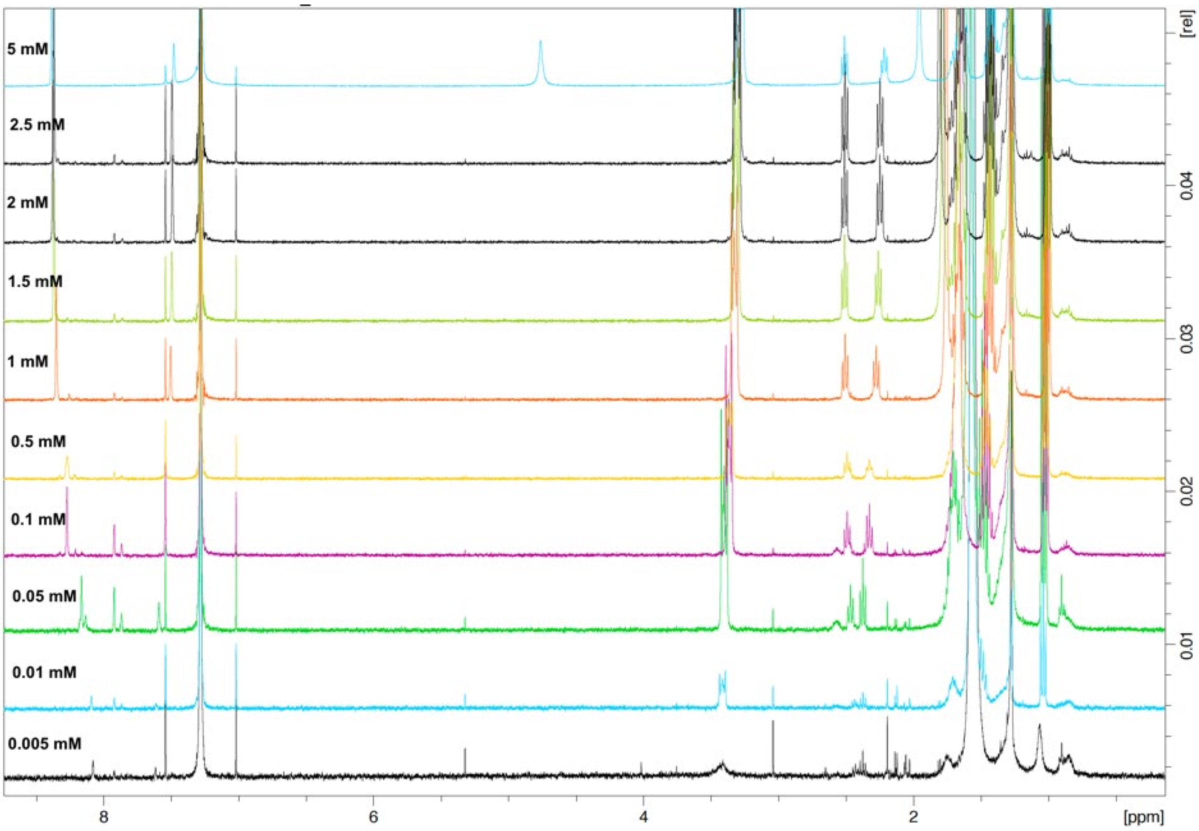

Figure S20: Concentration-dependent ^{1}H NMR titration spectra as a stack plot for aryl amide **3b** + TBAOH in $CDCl_3$ at 298 K. Dimerisation constant determined was 8483.76 M^{-1} and calculated by fitting the average chemical shifts of two aromatic C-H peaks to a NMR Dimer Aggregation model on bindfit with concentrations of **3b** between 5-0.005 mM.

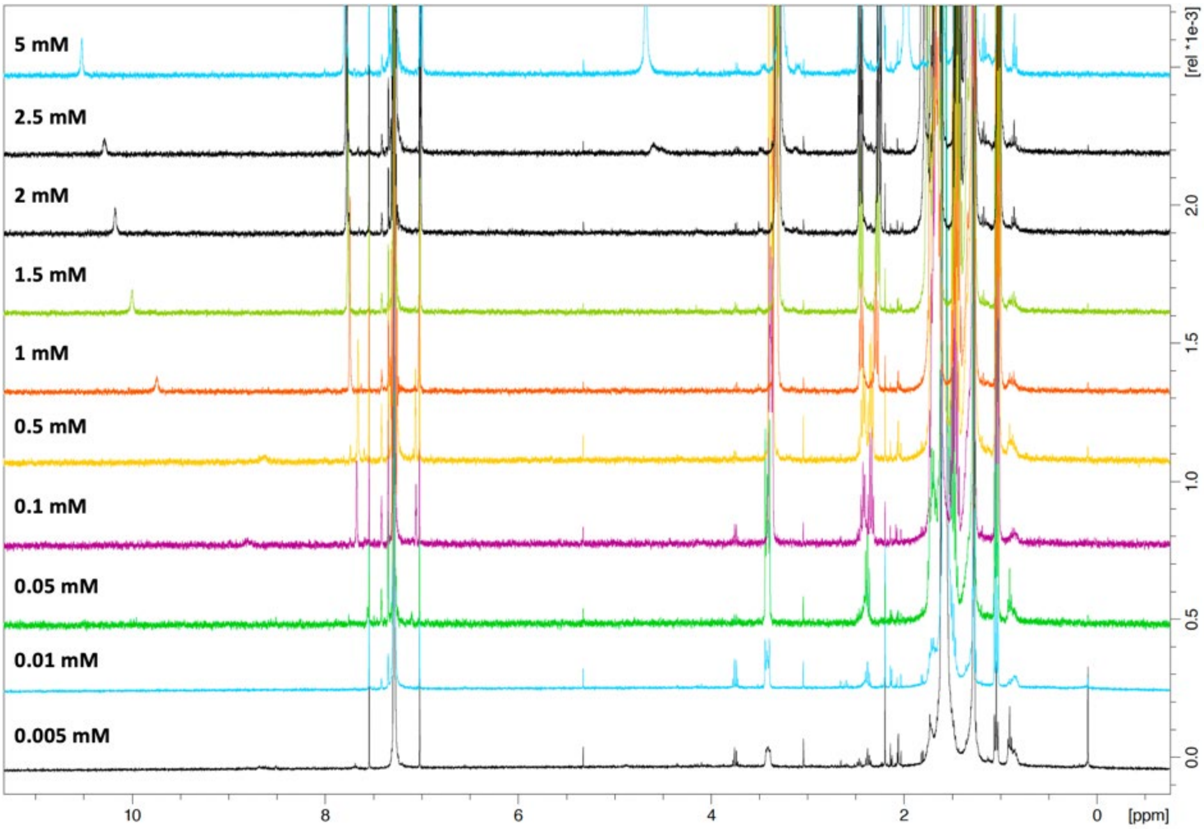

Figure S21: Concentration-dependent ^{1}H NMR titration spectra as a stack plot for aryl amide **4b** + TBAOH in $CDCl_3$ at 298 K. Dimerisation constant determined was 9297.37 M^{-1} and calculated by fitting the average chemical shifts of two aromatic C-H peaks to a NMR Dimer Aggregation model on bindfit with concentrations of **4b** between 5-0.005 mM.

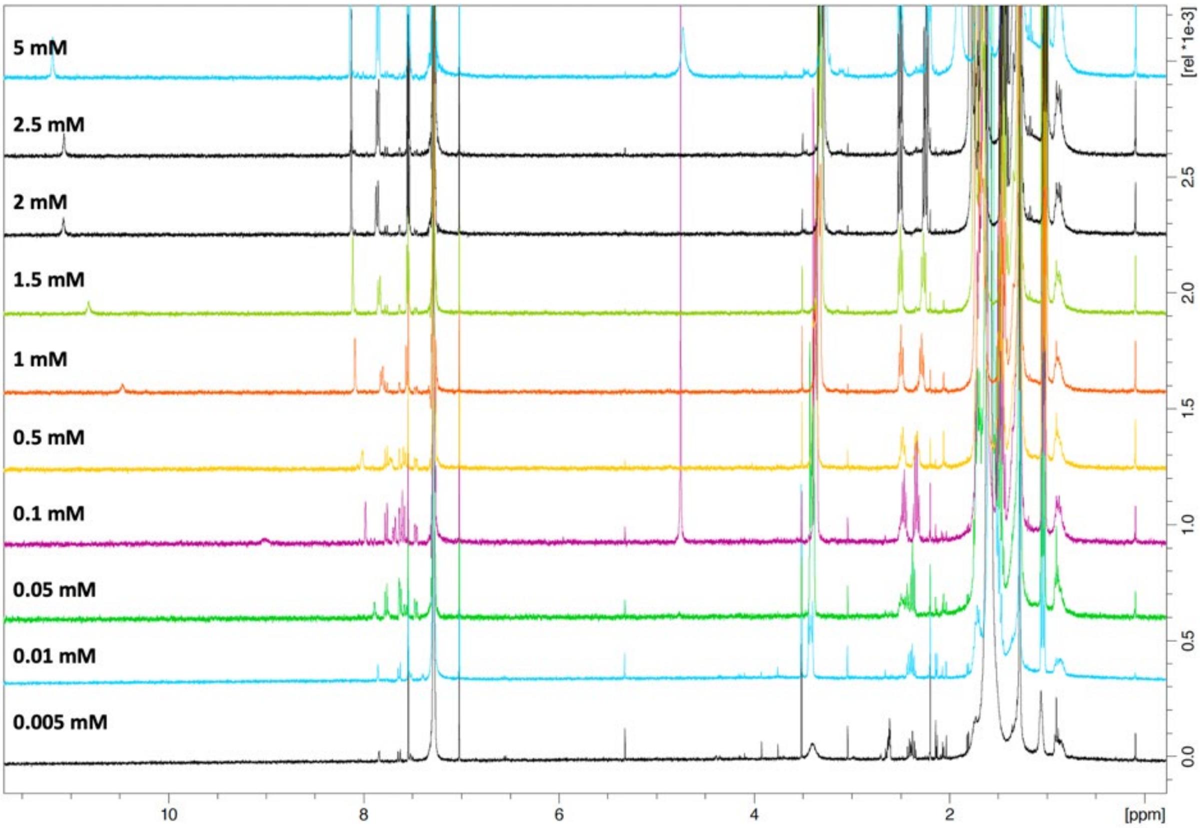

Figure S22: Concentration-dependent ^{1}H NMR titration spectra as a stack plot for aryl amide **5b** + TBAOH in $CDCl_3$ at 298 K. Dimerisation constant determined was 2825.96 M^{-1} and calculated by fitting the average chemical shifts of two aromatic C-H peaks to a NMR Dimer Aggregation model on bindfit with concentrations of **5b** between 5-0.005 mM.

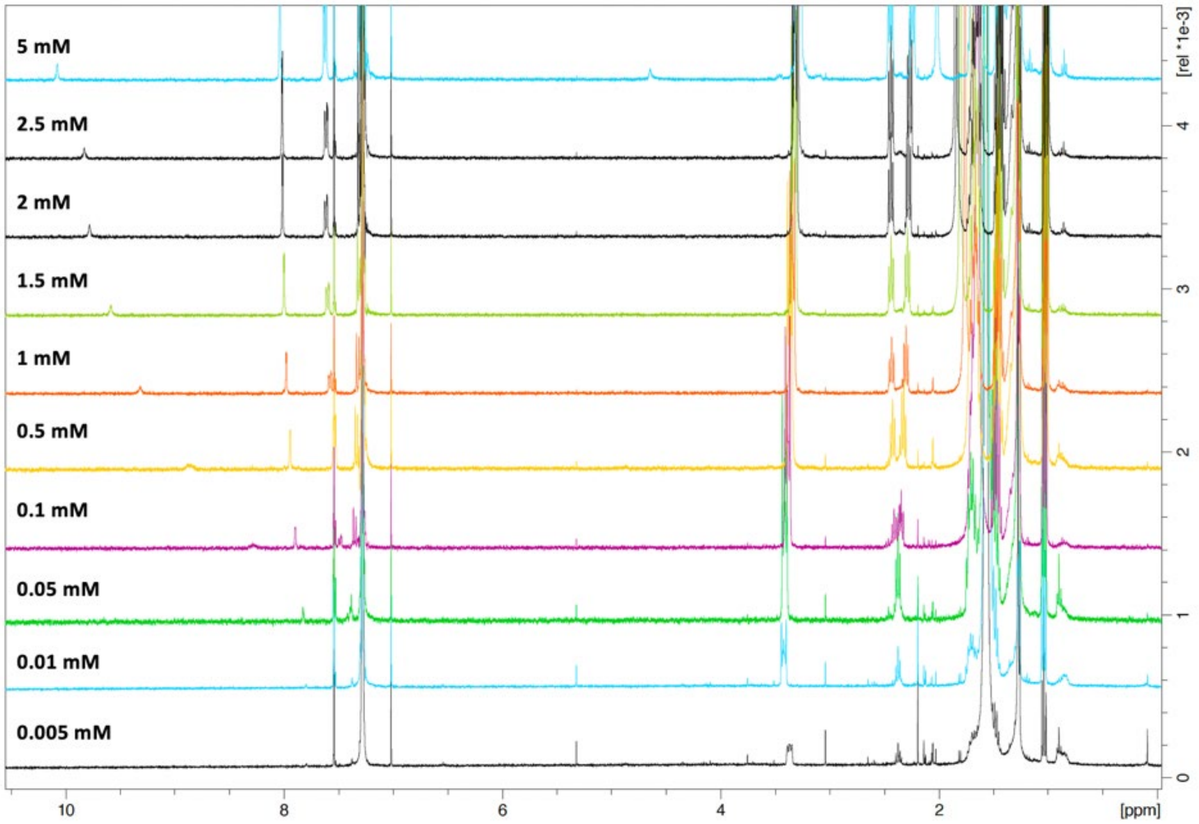

Figure S23: Concentration-dependent ^{1}H NMR titration spectra as a stack plot for aryl amide **6b** + TBAOH in $CDCl_3$ at 298 K. Dimerisation constant determined was 2366.56 M^{-1} and calculated by fitting the average chemical shifts of two aromatic C-H peaks to a NMR Dimer Aggregation model on bindfit with concentrations of **6b** between 5-0.005 mM.

6. Supporting Figures- Computational Evaluation

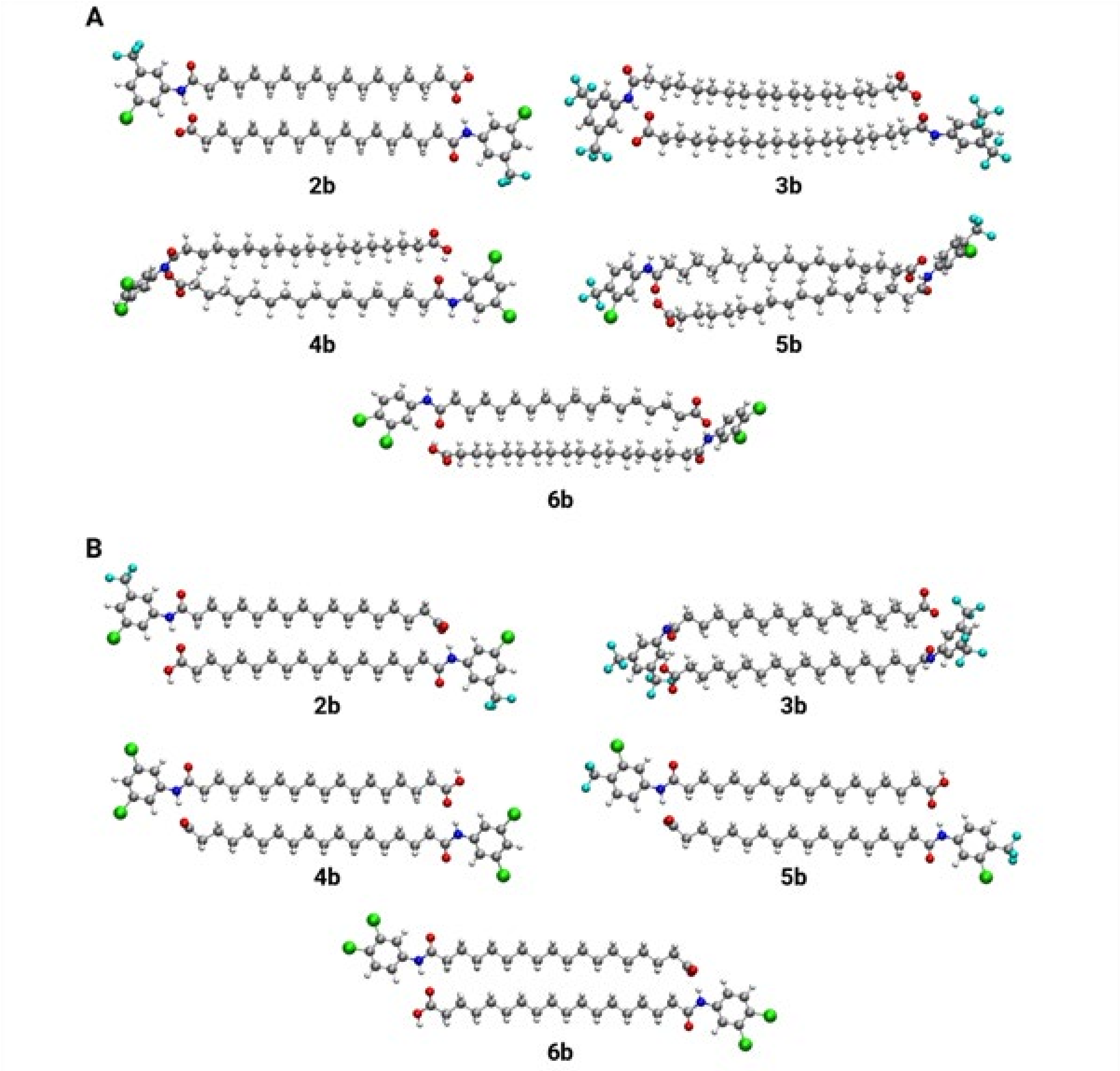

Figure S24: DFT optimized conformations of dimers solvated in benzene (A) and water (B). Optimizations were carried out using Gaussian 16 rev. C.01 at the M06-2X-D3/6-31G(d,p) level of theory.

7. Experimental Procedures – Chemistry

General chemistry

All chemical reagents, analytical grade solvents and aryl amide intermediates were purchased from Sigma Aldrich/Merck (Castle Hill, NSW, Australia) or Fluorochem (Derbyshire, United Kingdom). The purity of all tested compounds was confirmed to be ≥ 95% by absolute quantitative ^{1}H NMR (qNMR) spectroscopy. Reactions were monitored by thin-layer chromatography (TLC) using silica gel 60 F_{254} plates. TLC plates were visualised with UV light and potassium permanganate TLC stain. ^{1}H and ^{13}C NMR spectra were acquired using an Agilent or Bruker 400/500 MHz spectrometer in deuterated chloroform ($CDCl_3$) or DMSO-d_6 at 298 K. Melting point determination was performed using Stuart automatic melting point. High-resolution mass spectroscopy (HRMS) was performed on an Agilent 6510 Accurate-Mass Q-TOF Mass Spectrometer equipped with an ESI source.

Synthesis of 2b - 6b

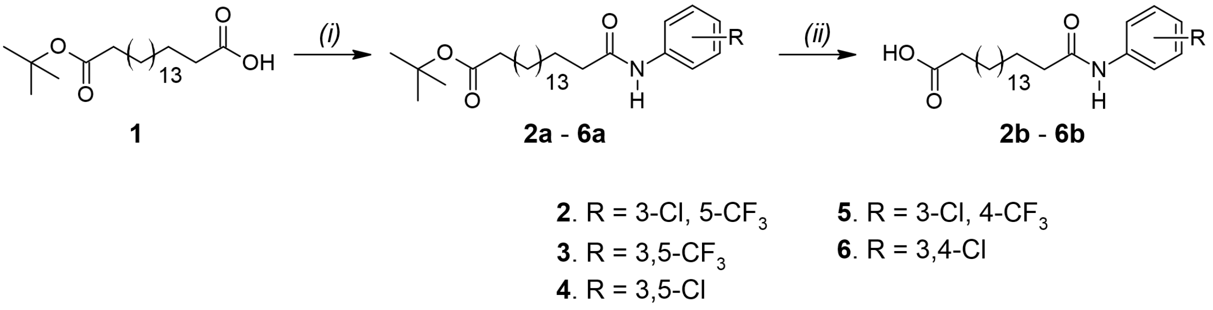

Scheme 1. Synthesis of arylamide substituted fatty acids

General Procedure A: COMU Coupling

1 (0.77 mmol, 200 mg), COMU (0.77 mmol, 332 mg) and Et_3N (1.54 mmol) were dissolved in anhydrous DMF (6 mL) under nitrogen gas and stirred for 5 min at room temperature. The appropriately substituted aniline (0.77 mmol) was added, and the reaction mixture was stirred at room temperature for 3 hrs. The reaction mixture was diluted with EtOAc (50 mL), and the organic layer was washed with 1 M HCl (2 x 25 mL), saturated $NaHCO_3$ (2 x 25 mL) and brine (2 x 10 mL). The organic layer was dried over Mg_2SO_4, and concentrated under reduced pressure. The crude products were purified using stepwise gradient elution on silica gel with DCM/EtOAc (100:0 to 98:2).

*Tert-butyl 18-((3-chloro-5-(trifluoromethyl)phenyl)amino)-18-oxooctadecanoate (**2a**)*

White solid (64%). ^{1}H NMR (400 MHz, $CDCl_3$): δ 7.90 (s, 1H), 7.67 (s, 1H), 7.39 (s, 1H), 7.35 (s, 1H), 2.39 (t, J = 7.4 Hz, 2H), 2.22 (t, J = 7.4 Hz, 2H), 1.75 (quint, J = 7.4 Hz, 2H), 1.62-1.58 (m, 2H), 1.47 (s, 9H), 1.39-1.27 (m, 24H). ^{13}C NMR (100 MHz, $CDCl_3$): δ 173.6, 171.8, 139.7, 135.4, 132.5 (q, J = 33 Hz), 123.1 (q, J = 271 Hz), 122.0, 120.8, 114.4 (q, J = 3 Hz), 80.1, 37.6, 35.7, 29.6, 29.5, 29.5, 29.5, 29.4, 29.4, 29.3, 29.3, 29.2, 29.1, 28.3, 28.1, 25.4, 25.2. HRMS (ESI) *m/z* $[M]^+$ calculated for $C_{29}H_{45}ClF_3NO_3$ = 548.3144, found 548.3113.

*Tert-butyl 18-((3,5-bis(trifluoromethyl)phenyl)amino)-18-oxooctadecanoate (**3a**)*

White solid (56%). ^{1}H NMR (400 MHz, $CDCl_3$): δ 8.05 (s, 2H), 7.59 (s, 2H), 2.40 (t, J = 7.4 Hz, 2H), 2.20 (t, J = 7.5 Hz, 2H), 1.74 (p, J = 7.4 Hz, 2H), 1.57-1.53 (m, 2H), 1.44 (s, 9H), 1.38-1.25 (m, 24H). ^{13}C NMR (100 MHz, $CDCl_3$): δ 173.7, 172.0, 139.5, 132.3 (q, J = 33 Hz), 127.2, 123.1 (q, J = 271 Hz), 119.3, 117.2 (q, J = 4 Hz), 80.1, 37.6, 35.7, 29.5, 29.4, 29.3, 29.3, 29.2, 29.1, 28.1, 25.3, 25.2. HRMS (ESI) *m/z* $[M]^+$ calculated for $C_{30}H_{45}F_6NO_3$ = 582.3376, found 582.3376.

*Tert-butyl 18-((3,5-dichlorophenyl)amino)-18-oxooctadecanoate (**4a**)*

White solid (42%). ^{1}H NMR (500 MHz, $CDCl_3$): δ 7.50 (d, J = 0.8 Hz, 2H), 7.08 (t, J = 1.2 Hz, 2H), 2.34 (t, J = 7.5 Hz, 2H), 2.20 (t, J = 7.5 Hz, 2H), 1.71 (quint, J = 7.5 Hz, 2H), 1.58-1.55 (m, 2H), 1.44 (s, 9H), 1.36-1.25 (m, 24H). ^{13}C NMR (125 MHz, $CDCl_3$): δ 173.6, 171.8, 139.8, 135.2, 124.0, 117.9, 80.0, 37.7, 35.7, 29.6, 29.6, 29.6, 29.4, 29.3, 29.3, 29.2, 29.1, 28.1, 25.4, 25.2. HRMS (ESI) *m/z* $[M]^+$ calculated for $C_{28}H_{45}Cl_2NO_3$ = 515.2876, found 515.2883.

*Tert-butyl 18-((3-chloro-4-(trifluoromethyl)phenyl)amino)-18-oxooctadecanoate (**5a**)*

White solid (53%). ^{1}H NMR (400 MHz, $CDCl_3$): δ 7.82 (d, J = 1.2 Hz, 1H), 7.60 (d, J = 9.0 Hz, 2H), 7.47 (dd, J = 8.0. 1.2 Hz, 2H), 7.35-7.31 (m, 1H), 2.37 (t, J = 7.5 Hz, 2H), 2.20 (t, J = 6.5 Hz, 2H), 1.73 (quint, J = 5.6 Hz, 2H), 1.58-1.54 (m, 2H), 1.44 (s, 9H), 1.37-1.25 (m, 24H). ^{13}C NMR (125 MHz, $CDCl_3$): δ 173.5, 171.6, 141.8, 133.1, 128.2 (q, J = 3 Hz), 123.7 (q, J = 33 Hz), 122.6 (q, J = 271 Hz), 121.5, 116.7, 80.0, 37.8, 35.7, 29.6, 29.5, 29.5, 29.4, 29.4, 29.3, 29.3, 29.2, 29.1, 28.1, 25.3, 25.1. HRMS (ESI) *m/z* $[M]^+$ calculated for $C_{29}H_{45}ClF_3NO_3$ = 548.3149, found 548.3113.

*Tert-butyl 18-((3,4-dichlorophenyl)amino)-18-oxooctadecanoate (**6a**)*

White solid (41%). ^{1}H NMR (500 MHz, $CDCl_3$): δ 7.77 (d, J = 0.8 Hz, 1H), 7.37-7.34 (m, 2H), 7.22 (s, 1H), 2.35 (t, J = 7.5 Hz, 2H), 2.20 (t, J = 7.5 Hz, 2H), 1.71 (quint, J = 7.5 Hz, 2H), 1.59-1.56 (m, 2H), 1.44 (s, 9H), 1.36-1.25 (m, 24H). ^{13}C NMR (125 MHz, $CDCl_3$): δ 173.4, 171.4, 137.4, 132.8, 130.4, 121.3, 118.8, 79.9, 37.7, 35.7, 29.6, 29.6, 29.6, 29.5, 29.4, 29.4, 29.3, 29.3, 29.2, 29.1, 28.1, 25.4, 25.1. HRMS (ESI) *m/z* $[M]^+$ calculated for $C_{28}H_{45}Cl_2NO_3$ = 514.2858, found 514.2849.

General Procedure B: Ester Hydrolysis

To a solution of the **2a-6a** (0.20 mmol) in dry DCM (4 mL) was added TFA (3 mL) dropwise, and the reaction mixture was stirred at room temperature for 3 hours. The resulting solution was diluted with EtOAc (20 mL), and the organic layer was washed with 0.5 M NaOH (25 mL), H_2O (25 mL) and 1% HCl (25 mL). The organic layer was dried with Mg_2SO_4, and the solvent was removed under reduced pressure to isolate the aryl-amides as white solids.

*18-((3-chloro-5-(trifluoromethyl)phenyl)amino)-18-oxooctadecanoic acid (**2b**)*

White solid (95%). M.P. = 124-126 °C. ^{1}H NMR (500 MHz, DMSO-d_6): δ 10.46 (s, 1H), 7.99 (s, 1H), 7.95 (s, 1H), 7.49 (s, 1H), 2.33 (t, J = 7.5 Hz, 2H), 2.17 (t, J = 7.5 Hz, 2H), 1.60-1.50 (m, 2H), 1.50-1.45 (m, 2H), 1.27-1.20 (m, 24H). ^{13}C NMR (100 MHz, DMSO-d_6): δ 174.9, 172.7, 141.9, 134.7, 131.5 (q, J = 33 Hz), 123.7 (q, J = 271 Hz), 122.2, 119.4 (q, J = 4 Hz), 114.2 (q, J = 4 Hz), 36.9, 34.1, 29.5, 29.5, 29.4, 29.4, 29.3, 29.2, 29.2, 29.0, 29.0, 25.2, 25.0. HRMS (ESI) *m/z* $[M]^+$ calculated for $C_{25}H_{37}ClF_3NO_3$ = 492.0144, found 492.0148. qNMR purity = 96.4 %

*18-((3,5-bis(trifluoromethyl)phenyl)amino)-18-oxooctadecanoic acid (**3b**)*

White solid (92%). M.P. = 102-104 °C. ^{1}H NMR (500 MHz, DMSO-d_6): δ 11.93 (s, 1H), 10.52 (s, 1H), 8.26 (s, 2H), 7.72 (s, 1H), 2.35 (t, J = 7.5 Hz, 2H), 2.16 (t, J = 7.5 Hz, 2H), 1.55-1.50 (m, 2H), 1.50-1.45 (m, 2H), 1.27-1.21 (m, 2H). ^{13}C NMR (100 MHz, DMSO-d_6): δ 175.0, 172.9, 141.6, 131.2 (q, J = 33 Hz), 123.7 (q, J = 271 Hz), 119.0, 116.1, 36.9, 34.1, 29.5, 29.5, 29.4, 29.4, 29.3, 29.2, 29.2, 29.0, 29.0, 25.2, 25.0. HRMS (ESI) *m/z* $[M]^+$ calculated for $C_{26}H_{37}F_6NO_3$ = 525.5673, found 525.5675. qNMR purity = 98.6 %

*18-((3,5-dichlorophenyl)amino)-18-oxooctadecanoic acid (**4b**)*

White solid (88%). M.P. = 120-124 ˚C. ^{1}H NMR (500 MHz, DMSO-d_6): δ 11.93 (s, 1H), 10.19 (s, 1H), 7.65 (s, 2H), 7.23 (s, 1H), 2.30 (t, *J* = 7.5 Hz, 2H), 2.18 (t, *J* = 7.5 Hz, 2H), 1.58-1.55 (m, 2H), 1.48-1.45 (m, 2H), 1.26-1.22 (m, 24H). ^{13}C NMR (100 MHz, DMSO-d_6): δ 174.9, 172.5, 142.1, 134.5, 122.6, 117.5, 36.9, 34.1, 29.5, 29.5, 29.4, 29.4, 29.3, 29.2, 29.2, 29.0, 29.0, 25.3, 25.0. HRMS (ESI) *m*/*z* [M]$^+$ calculated for $C_{24}H_{37}Cl_2NO_3$ = 458.4615, found 458.4611. qNMR purity = 98.5 %

*18-((3-chloro-4-(trifluoromethyl)phenyl)amino)-18-oxooctadecanoic acid (**5b**)*

White solid (93%). M.P. = 122-125 ˚C. ^{1}H NMR (400 MHz, $CDCl_3$): δ 10.40 (s, 1H), 8.03 (d, *J* = 2.0 Hz, 1H), 7.80 (d, *J* = 11.0 Hz, 1H), 7.65 (dd, *J* = 11.0, 1.5 Hz, 1H), 2.32 (t, *J* = 7.5 Hz, 2H), 2.18 (t, *J* = 7.5 Hz, 2H), 1.60-1.57 (m, 2H), 1.49-1.46 (m, 2H), 1.40-1.20 (m, 24H). ^{13}C NMR (125 MHz, DMSO-d_6): δ 174.9, 169.0, 144.0, 123.3, 128.8, 128.1 (q, *J* = 3 Hz), 125.2 (q, *J* = 31 Hz), 123.4 (q, *J* = 271 Hz), 41.9, 39.1, 34.1, 29.5, 29.5, 29.4, 29.4, 29.4, 29.2, 29.2, 29.0, 26.8, 24.9. HRMS (ESI) *m*/*z* [M]$^+$ calculated for $C_{25}H_{37}ClF_3NO_3$ = 492.0144, found 492.0147. qNMR purity = 98.2 %

*18-((3,4-dichlorophenyl)amino)-18-oxooctadecanoic acid (**6b**)*

White solid (93%). M.P. = 118-120 ˚C. ^{1}H NMR (500 MHz, DMSO-d_6): δ 11.93 (s, 1H), 10.14 (s, 1H), 7.99 (s, 1H), 7.52 (d, *J* = 9.0 Hz, 1H), 7.48-7.45 (dd, *J* = 9.0, 2.5 Hz, 1H), 2.30 (t, *J* = 7.5 Hz, 2H), 2.17 (t, *J* = 7.5 Hz, 2H), 1.58-1.55 (m, 2H), 1.48-1.45 (m, 2H), 1.26-1.22 (m, 24H). ^{13}C NMR (120 MHz, DMSO-d_6): δ 174.9, 172.3, 139.3, 131.4, 131.1, 124.7, 120.6, 119.4, 36.9, 34.1, 29.5, 29.5, 29.4, 29.4, 29.3, 29.2, 29.2, 29.0, 25.3,25.0. HRMS (ESI) *m*/*z* [M]$^+$ calculated for $C_{25}H_{37}ClF_3NO_3$ = 492.0144, found 492.0142. qNMR purity = 97.1 %

Synthesis of 5b-C12

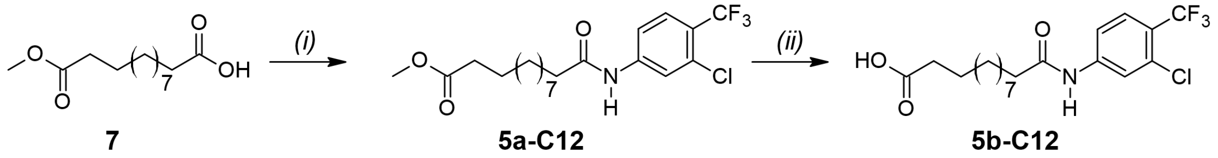

Scheme S2: Synthesis of **5b-C12**, a chain shortened analogue of **5b**

*Methyl 12-((3-chloro-4-(trifluoromethyl)phenyl)amino)-12-oxododecanoate (**5a-C12**)*

Prepared following General Procedure A where **1** was replaced with 12-methoxy-12-oxododecanoic acid (**7**). White solid (55%). ^{1}H NMR (500 MHz DMSO-d_6): δ 8.00 (d, J = 8.5 Hz, 1H), 7.96 (s, 1H), 7.81 (d, J = 8.6 Hz, 1H), 7.46 (s, 1H), 3.69 (s, 3H), 2.43 (t, J = 7.5 Hz, 2H), 2.33 (t, J = 7.5 Hz, 2H), 1.76 (quint, J = 7.5 Hz, 2H), 1.62 (quint, J = 7.5 Hz, 2H), 1.45-1.25 (m, 12H). ^{13}C NMR (100 MHz, $CDCl_3$): δ 174.6, 171.9, 141.9, 133.0, 133.0, 128.2 (q, J = 5 Hz), 123.4 (q, J = 32 Hz), 122.8 (q, J = 271 Hz), 121.6, 116.8, 51.5, 37.3, 34.1, 29.2, 29.2, 29.1, 29.0, 25.3, 24.9. HRMS (ESI) m/z $[M]^+$ calculated for $C_{20}H_{27}ClF_3NO_3$ = 422.1704, found 422.1703.

*12-((3-chloro-4-(trifluoromethyl)phenyl)amino)-12-oxododecanoic acid (**5b-C12**)*

5a-C12 (0.10, 0.042g) mmol) was dissolved in ethanol (6 mL). 1M NaOH (2 mL) was added dropwise and the reaction mixture was stirred for 3 hours at 40 ˚C. Ethanol was removed under reduced pressure and the residue was acidified with 0.5 M HCl. The aqueous layer was extracted with chloroform (3 x 15 mL) and the combined organic layers were dried over Mg_2SO_4. Chloroform was removed under reduced to yield 0.037g of **5b-C12** as a white solid (92%). M.P. = 113-115 ˚C. ^{1}H NMR (500 MHz DMSO-d_6): δ 11.93 (s, 1H), 10.39 (s, 1H), 8.03 (d, J = 2.0 Hz, 1H), 7.77 (d, J = 11.0 Hz, 1H), 7.64 (dd, J = 11.0, 2.0 Hz, 1H), 2.34 (t, J = 7.5 Hz, 2H), 2.18 (t, J = 8.0 Hz, 2H), 1.60-1.57 (m, 2H), 1.50-1.47 (m, 2H), 1.27-1.24 (m, 12H). ^{13}C NMR (125 MHz, DMSO-d_6): δ 175.0, 172.4, 139.2, 132.5, 127.1 (q, J = 30 Hz), 124.1, 123.9, 132.2 (q, J = 271 Hz), 118.0 (q, J = 5 Hz), 36.8, 34.2, 29.3, 29.3, 29.2, 29.2, 29.1, 29.0, 25.3, 25.0. HRMS (ESI) m/z $[M]^+$ calculated for $C_{19}H_{25}ClF_3NO_3$ = 407.1475, found 407.1477. qNMR purity = 96.5 %

8. Experimental Procedures – Cell Culture

General Cell Culture

The human MDA-MB-231 triple-negative breast cancer cell line was obtained from ATCC (Manassas, VA). Cells were grown at 37°C in a humidified atmosphere of 5% CO2 in air in DMEM supplemented with 10% fetal bovine serum (Thermo Fisher Scientific) and 1% penicillin/streptomycin (Invitrogen). Confluent cells (80-90%) were harvested using Trypsin/EDTA after washing in PBS. Test compounds were prepared as DMSO stock solutions and diluted in cell media before cell treatment (final DMSO concentration 0.1%); control cells were treated with 0.1% DMSO alone.

MTS Assay

Cells were seeded on 96 well plates (5 x 10^3 cells per well) for 24 hours. The complete media was then replaced with serum-free media and left to incubate for 18 hours. The cells were then treated with various concentrations of the test compounds for 24 hours. Cell Titer AQ One solution (Promega, USA) was added to each well. Cells were incubated for 3 hrs at 37 ˚C, and absorbance was measured at 490 nm using a Tecan Infinite M1000 Pro plate reader.

JC-1 Assay

Cells were seeded on 96 well plates (1.5 x 10^4 cells per well) and allowed to adhere overnight. After serum removal for 24 hours the cells were treated with the test compounds at varying concentrations for 1 hour. Cells were incubated with JC-1 dye (Promega, USA) for 20 minutes and then washed twice with PBS. The fluorescence of JC-1 aggregates and monomers was measured at excitation/emission wavelengths of 535/595 nm and 485/535 nm, respectively. IC_{50} concentrations were defined as the drug concentration that shifted the JC-1 red/green fluorescence ratio by 50% (relative to the vehicle control) and was determined using non-linear regression analysis with Prism 7.0 (GraphPad Software, CA, USA).

ATP assay

Cells were seeded on 96 well plates (7.5 x 10^3 cells per well) and allowed to adhere overnight. After serum removal for 24 hours the cells were treated with the test compounds (20 μM) for 1-6 hours. Cells were then incubated with Cell Titer Glo solution (Promega, USA), mixed on an orbital shaker for two minutes, left at room temperature in dark conditions for 10 minutes and luminescence was read on a Tecan Infinite M1000 Pro plate reader.

Seahorse Assay

Mitochondrial function was measured by determining the OCR of cells with a Seahorse XF24 extracellular flux analyser (Seahorse Bioscience, MA, USA) according to the manufacturer's protocol. MDA-MB-231 cells were seeded in a 24-well XF cell culture microplate (2.5 x 10^4 cells per well) and allowed to adhere for 24 hours. The culture media was then replaced with XF Base Medium buffered to pH = 7.4 with 5mM HEPES and supplemented with 2 mM L-glutamine, 10 mM glucose and 2 mM sodium pyruvate. The cells were incubated at 37 ˚C without CO_2 for one hour then OCR was measured using an XF Cell Mito Stress Test Kit (Seahorse Bioscience, MA, USA). Aryl amides (40 µM), CCCP (5 µM) and DNP (20 µM) were loaded into the sensor cartridge and OCR was measured using a modified cycling program.

LDH Assay

Cells were seeded on 96 well plates (5 x 10^3 cells per well) and allowed to adhere overnight. After serum removal for 24 hours the cells were treated with the test compounds (40 µM) for 6 hours. Well media was homogenised, aliquots were added to LDH storage buffer and frozen until analysis. Maximum LDH release was determined by treating cells with Triton X-100 for 15 minutes. These solutions containing media were frozen at -20 ˚C until the day of the assay. Samples were thawed, incubated with LDH detection reagent at room temperature for one hour and luminescence was measured on a Tecan infinite M1000 Pro plate reader.

Statistical Analysis

Data are presented as mean ± standard error (SEM) from three independent experiments (N=3) with at least 2 internal replicates. Dose-response curves were constructed using log(inhibitor) vs response, variable slope (4 parameters) non-linear regressions on GraphPad Prism 8. Absolute IC_{50} concentrations were interpolated from these normalized curves (data normalized to DMSO vehicle control) with the top constrained to 100%. ATP data are expressed as mean percentage of time-matched DMSO control. (*) $P < 0.05$, (**) $P < 0.01$, (***) $P < 0.001$ versus time-matched control by two-way ANOVA with Dunnett's multiple comparison test. Seahorse data normalized to baseline OCR prior to oligomycin addition.

9. Experimental Procedures – HPTS Assay

The HPTS Assay was performed as previously described.[3] HPTS assays were conducted using POPC LUVs (200 nm diameter) vesicles were loaded with an internal solution containing pH-sensitive fluorescent dye HPTS (1 mM), HEPES buffer (10 mM) and potassium gluconate (100 mM). An external solution of HEPES buffer (10 mM) and potassium gluconate (100 mM) was also prepared, and both solutions were buffered to pH 7.

Unilamellar vesicles were prepared following a procedure outlined previously by Gale *et al*. A chloroform solution of POPC (37.5 mM, 4 mL) was transferred to a pre-weighed round-bottom flask, and the solvent was removed using a rotary evaporator. The pressure was lowered slowly to ensure the formation of a smooth lipid film. Subsequently, the film was dried in vacuo for 4-24 h, and the mass of lipid was recorded. The lipids were rehydrated with 4 mL of internal solution (this number should correspond to the volume of POPC solution used initially) and vortexed until all lipids were removed from the sides of the flask and were suspended in solution. The lipids were subjected to 9 cycles of freeze-thaw by freezing using a dry ice/acetone bath and thawing in lukewarm water. Following this, the vesicles were left to rest at room temperature for 30 min. The lipids were extruded through a 200 nm polycarbonate membrane 25 times to form monodisperse vesicles. Only 1 mL of solution was extruded at a time before being collected. Finally, any residual unencapsulated salt from the internal solution was using a B19 column packed with hydrated G-25 Sephadex®, which had been pre-saturated with the respective external solution. The lipid suspensions were diluted with the external solution to afford a stock solution (10 mL) of a known concentration.

HPTS Assay

For a given experiment, the prepared vesicles were diluted to a concentration of 0.1 mM in a 4.5 mL plastic cuvette. A pH gradient is required to drive transport through the vesicle membrane in these experiments before the transporter is added. An aliquot of aqueous NaOH solution (25 μL, 0.5 M) was added to increase the pH of the external solution by approx. one pH unit to pH 8.0. Following this, valinomycin (5 μL of 25 μM DMSO solution, 0.05 mol%) was added to each cuvette. Transport was initiated with the addition of the transporter as a DMSO solution (5 μL) and ended with the addition of detergent (Triton X-100 (10% v/v in water), 25 μL) at t = 210 s to lyse the vesicles, and a final fluorescence intensity reading was recorded at t = 300 s to signify 100% proton efflux.

Dose-Response Hill Analysis

The changes in the fluorescent activity of intravesicular HPTS were used to detect pH changes during the experiments, and hence represent proton efflux. The acidic and basic forms of the HPTS probe were excited at λ_{ex} = 403 nm and λ_{ex} = 460 nm, respectively, and the fluorescence emission of both forms recorded at λ_{em} = 510 nm. The intensity ratio of basic form to acidic form was determined, and the fractional fluorescence intensity (I_F) was calculated using the equation:

$$I_f = \frac{R_t - R_0}{R_d - R_0}$$

Where R_t is the ratiometric fluorescence value at a given time (t), R_0 is the ratiometric fluorescence value at t = 0 s and R_d is the fluorescence ratiometric value recorded at t = 280 s following the addition of detergent.

Dose-response experiments were performed at a minimum of five transporter concentrations plus a blank DMSO control run. I_F was plotted as a function of transporter concentration (mol%, with respect to lipid concentration). The I_F value at t = 200 s for each tested transporter concentration was fit to an adapted Hill Equation, using Origin 2021b (Academic), given as:

$$y = y_0 + (y_{max} - y_0)\frac{x^n}{k^n + x^n}$$

where y_0 is the I_F value at t = 200 s for the DMSO blank run, y_{max} is the maximum I_F value, n is the Hill coefficient, and k is a derived parameter. A derived equation was used to calculate the EC_{50} value, the transporter concentration required to facilitate 50% chloride efflux, given as:

$$EC_{50} = k(\frac{0.5}{y_1 - y_0})^{1/n}$$

where k and n are the derived parameters from the Hill equation, y_0 is the percentage chloride efflux at t = 0 s, and y_1 is the percentage chloride efflux at t = 280 s.

Incubation Studies

Additional experiments were conducted to assess the effect of protonophore incubation on the rate of proton efflux. Experiments were completed for each compound at the same loading of protonophore (0.5 mol%) and performed under two conditions. (1) Base addition first, followed by protonophore addition to initiate the experiment (t = 0 s incubation). (2) Protonophore

addition first, followed by base addition to initiate the experiment after a t = 300 s interval (t = 300 s incubation). The efflux plots of these experiments were depicted on the same axes for qualitative comparison. The initial rates and incubation enhancement factors were calculated to provide a quantitative analysis.

10. Experimental Procedures – NMR Studies

Anion Binding ^{1}H NMR Studies

Aryl amide esters **2a-6a** were dissolved in acetone-d_6 subject to ^{1}H NMR measurements on a Bruker AVANCE III 400 NMR Spectrometer. TBA-OAc in acetone-d_6 was sequentially added to the NMR tube, varying in concentrations of 0.1 – 30 mM. The chemical shift of the N-H peak at various concentrations was fitted to a 1:1 binding model to retrieve anion binding constants.[4]

Concentration-Dependent ^{1}H NMR Studies

A suspension of aryl amides **2b-6b** in $CDCl_3$ was treated with 1.0 equivalent of tetrabutylammonium hydroxide (TBAOH, ~40% in H_2O) and sonicated for 20 min to give a clear solution of (**2b-6b**)-TBAOH at 5 mM. The solution was diluted to various concentrations in $CDCl_3$ and subject to ^{1}H NMR measurements on a Bruker AVANCE III 400 NMR Spectrometer. The chemical shifts of two aromatic C-H peaks at various concentrations were fitted to a monomer-dimer equilibrium model to retrieve dimerisation constants.[5, 6]

11. Experimental Procedures - Computational Evaluation

Both truncated and complete aryl amide structures were constructed using GaussView 6.[7] Aryl amide dimers were constructed using the optimized monomeric structures. All DFT calculations performed using Gaussian 16, revision C.01[8] at the M062x-D3/6-31G(d, p)//M062X-D3/6-311++G(2df,2p) level of theory. Implicit solvation in benzene (e=2.2706) and water (e=78.3553) was carried out using the SMD model for all calculations.

For calculating dipole angle of aryl amides **1**, **4** and **5**, the probability of the formation of their two possible conformers were calculated using a Boltzmann Distribution given as:

$$p_i = \frac{1}{Q}\exp\left(-\frac{\varepsilon_i}{kT}\right) = \frac{\exp\left(-\frac{\varepsilon_i}{kT}\right)}{\sum_{j=1}^{M}\exp\left(-\frac{\varepsilon_j}{kT}\right)}$$

Where p_i is the probability of formation of the conformer, T is the temperature (298 K), k is typically a Boltzmann constant but in this case it is the gas constant R (0.00831 kJ/mol·K) and ε_i is the energy states of the conformer. The average bond angle was calculated by addition of the bond angles of each conformer when considering their probabilities:

$$\theta_{average} = (\theta_1 \times p_1) + (\theta_2 \times p_2)$$

Where $\theta_{average}$ is the average dipole angle for that aryl amide, θ_1 and θ_2 are the dipole angles of the conformers and p_1 and p_2 are the probabilities of the formation of each conformer.

12. NMR Characterisation

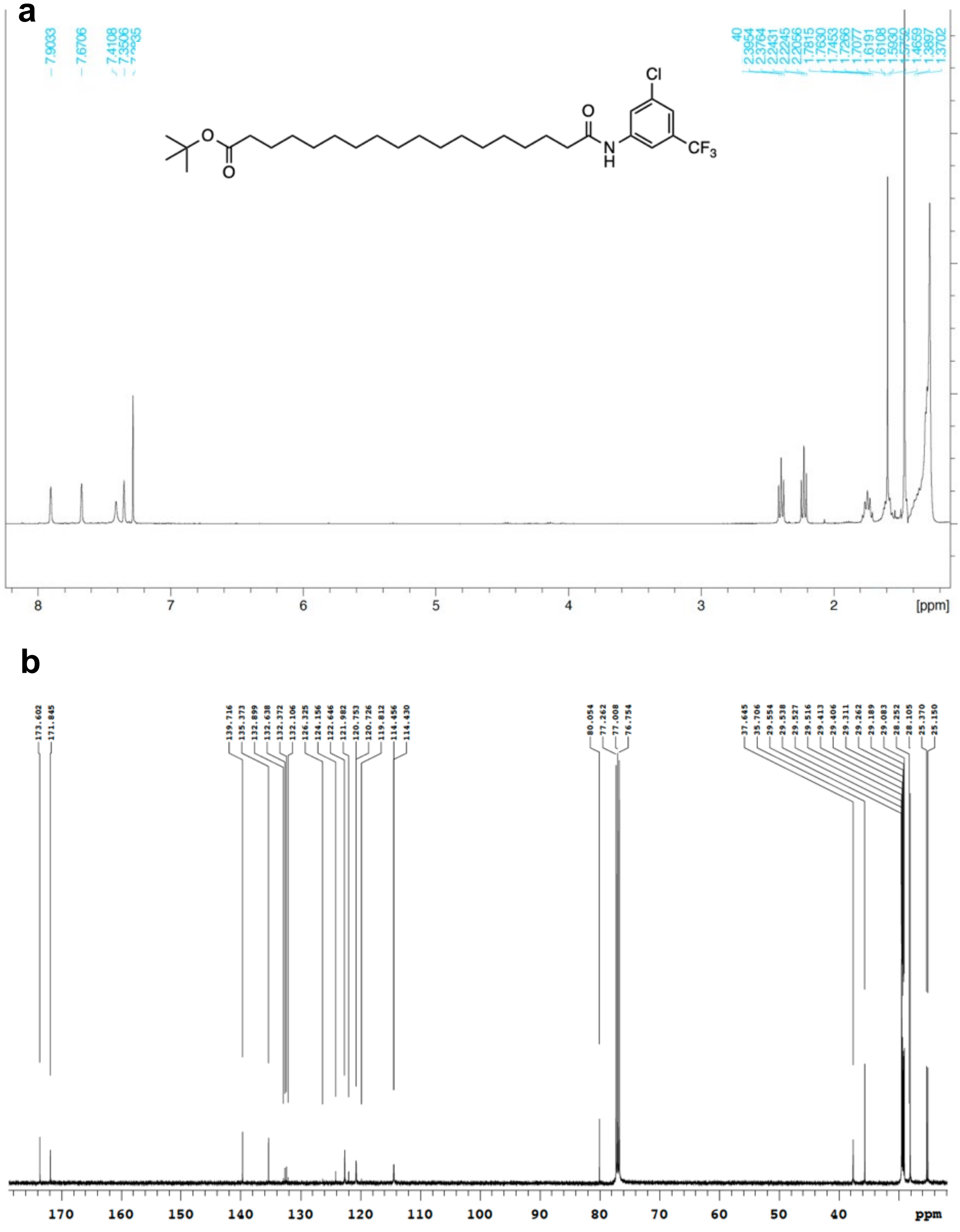

Figure S24: a) ^{1}H NMR spectrum of **2a** (400 MHz, $CDCl_3$). **b)** ^{13}C NMR spectrum of **2a** (125 MHz, $CDCl_3$)

a

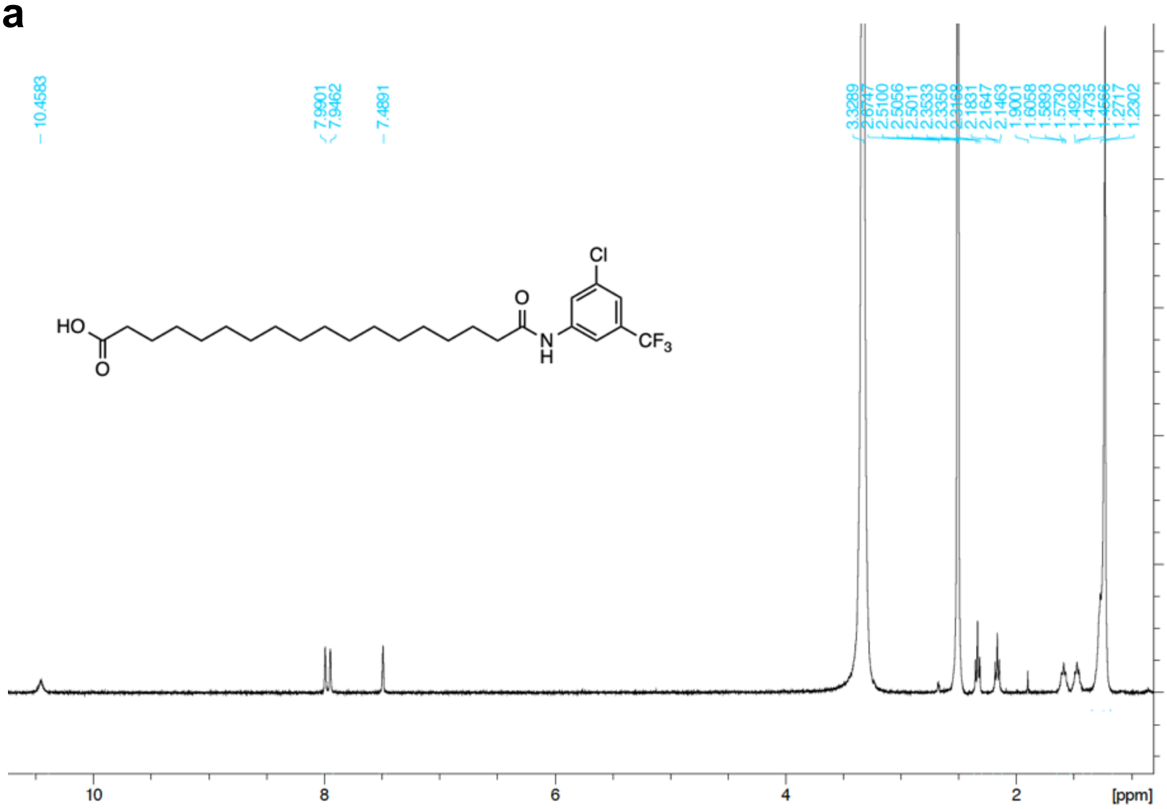

b

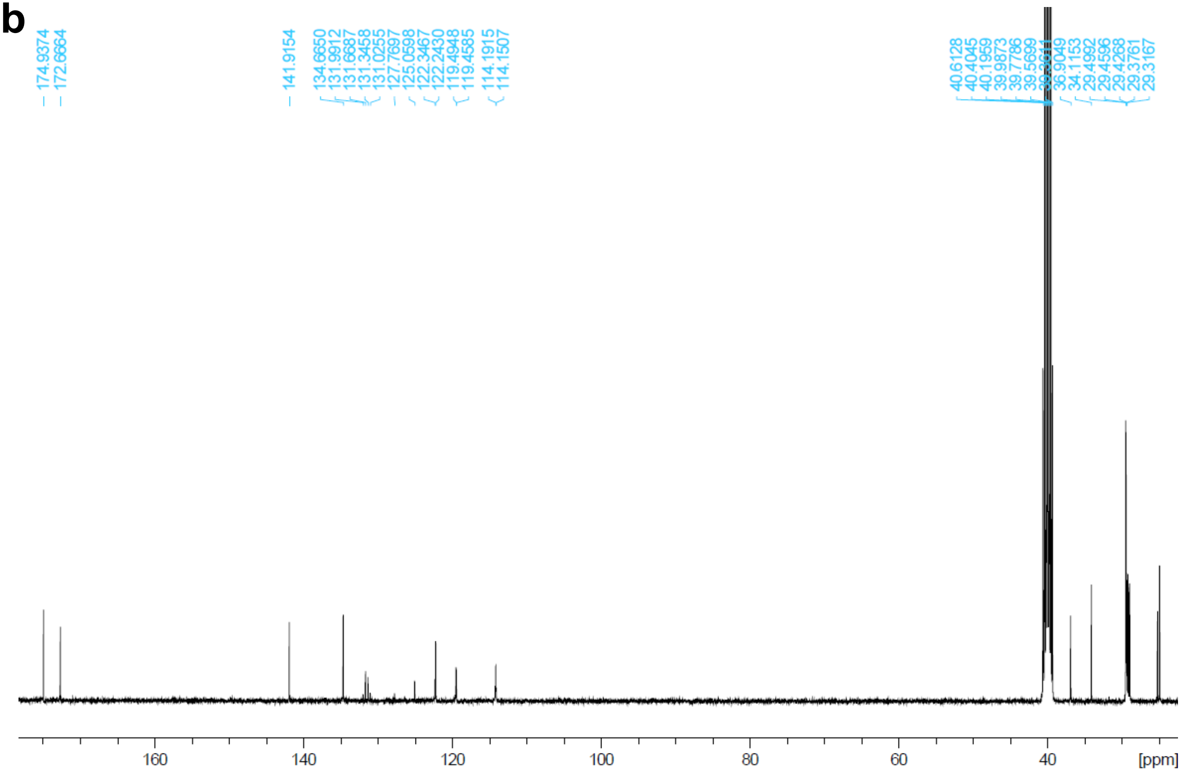

Figure S25: a) ^{1}H NMR spectrum of **2b** (500 MHz, DMSO-d_6). **b)** ^{13}C NMR spectrum of **2b** (100 MHz, DMSO-d_6).

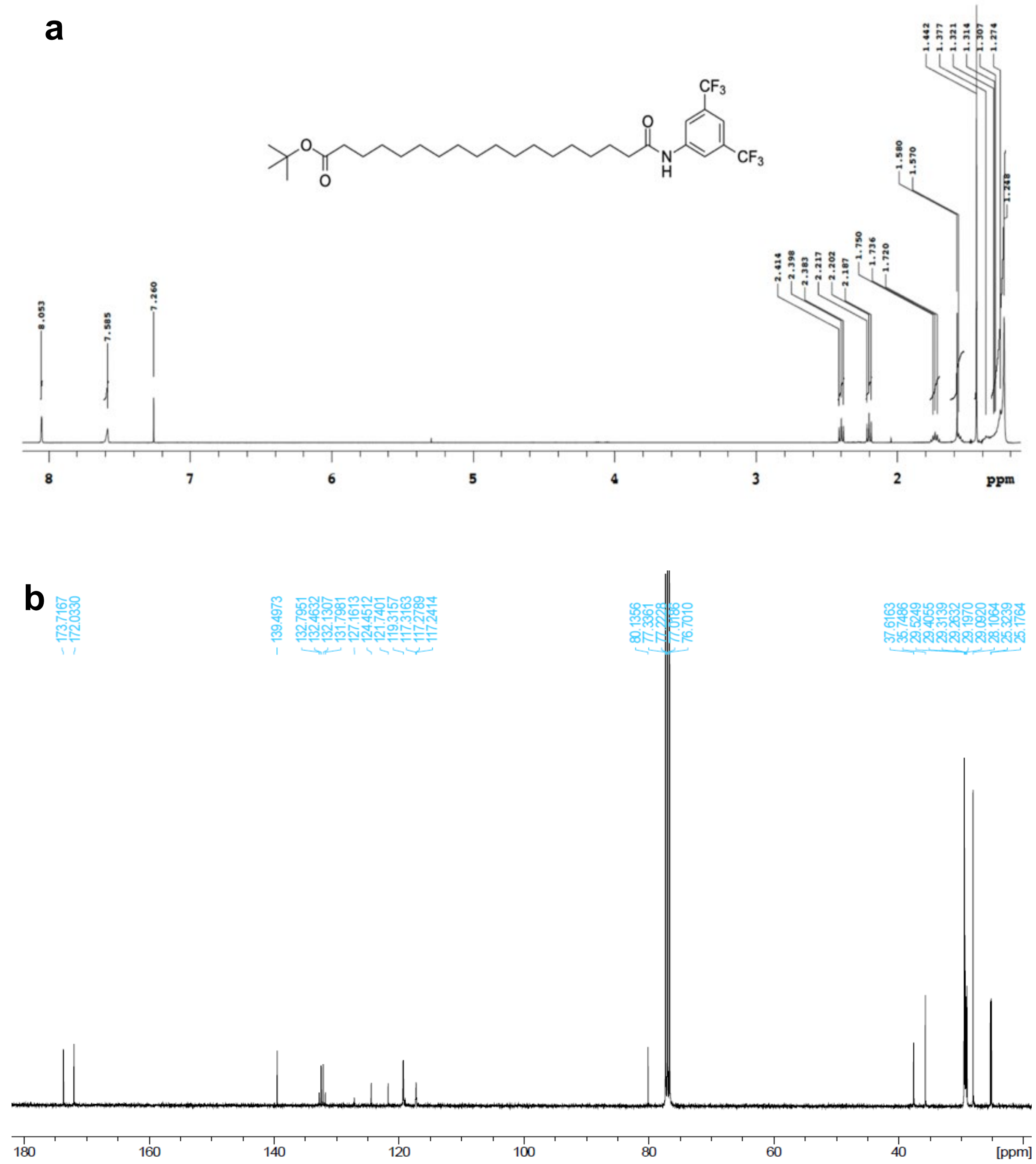

Figure S26: a) ^{1}H NMR spectrum of **3a** (400 MHz, $CDCl_3$). **b)** ^{13}C NMR spectrum of **3a** (100 MHz, $CDCl_3$).

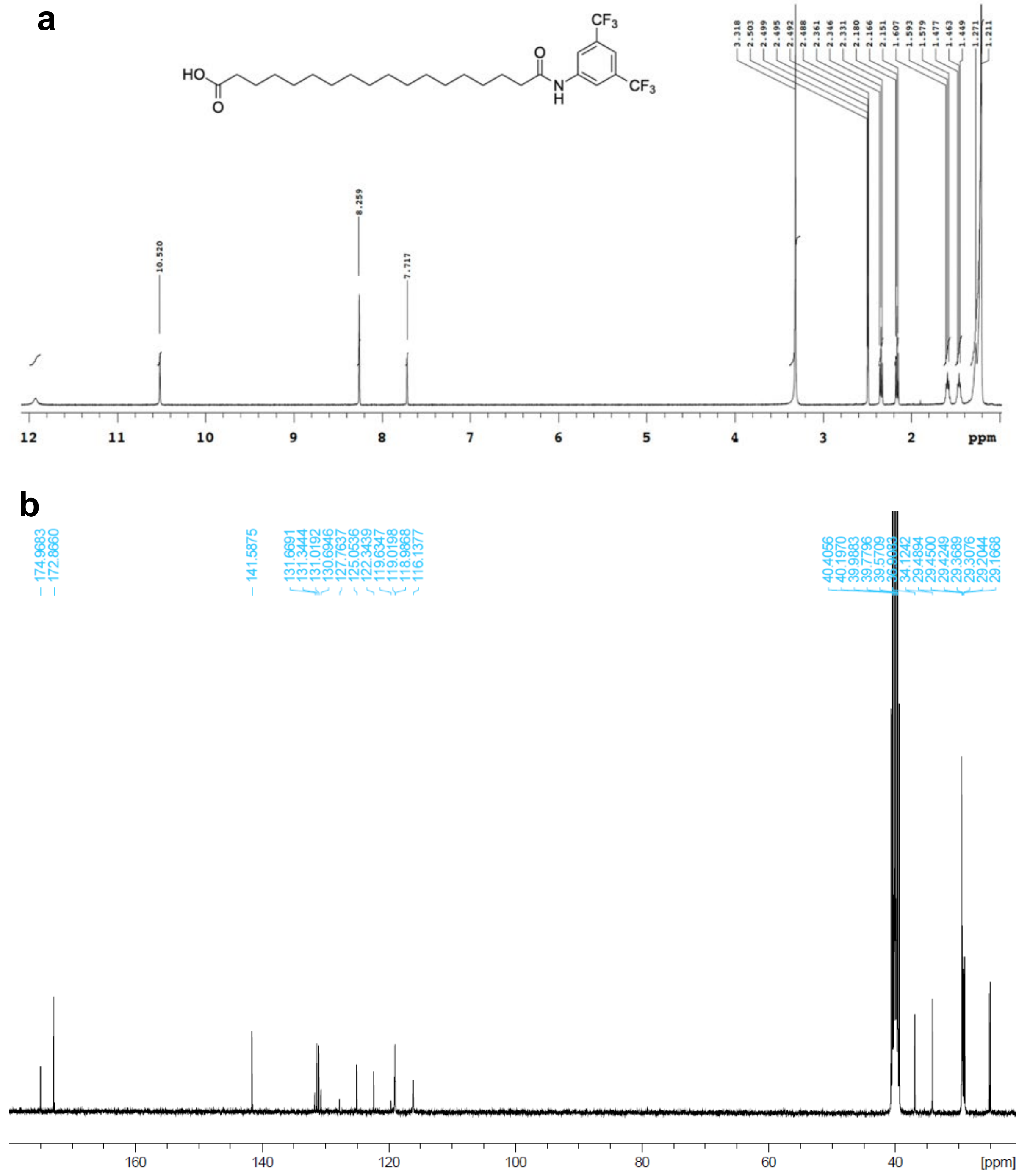

Figure S27: a) ^{1}H NMR spectrum of **3b** (500 MHz, DMSO-d_6). **b)** ^{13}C NMR spectrum of **3b** (100 MHz, DMSO-d_6).

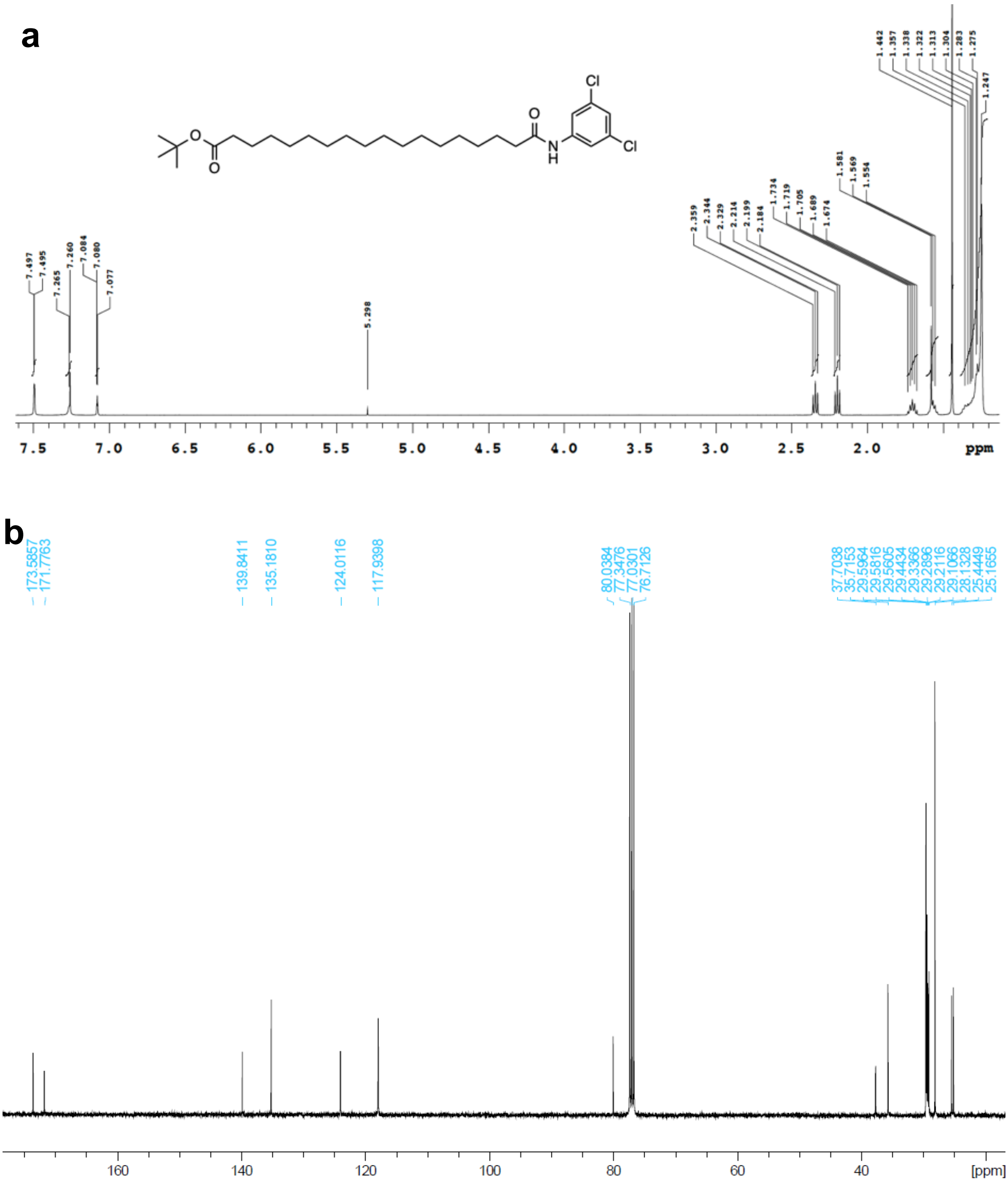

Figure S28: a) ^{1}H NMR spectrum of **4a** (400 MHz, $CDCl_3$). **b)** ^{13}C NMR spectrum of **4a** (125 MHz, $CDCl_3$).

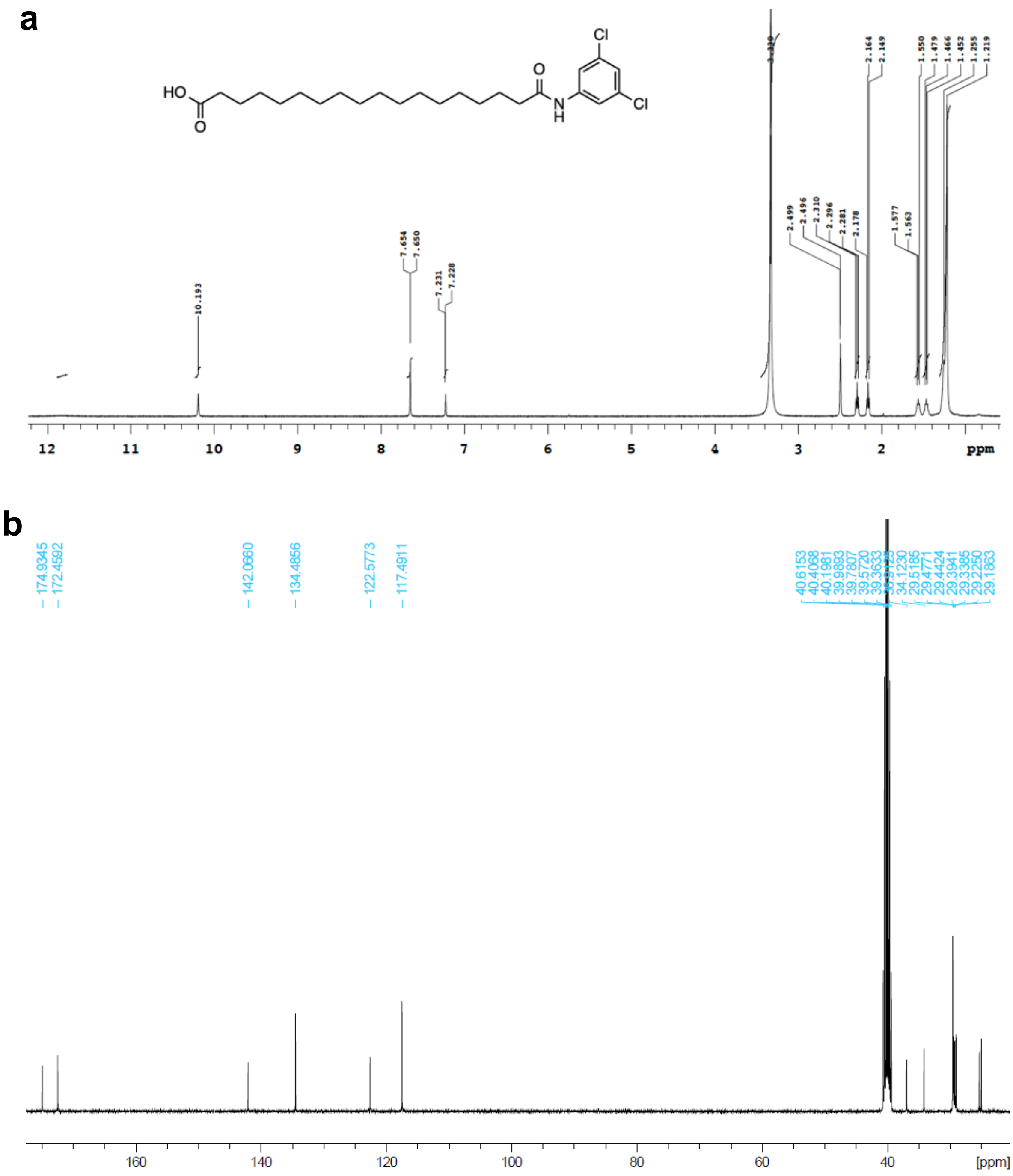

Figure S29: a) ^{1}H NMR spectrum of **4b** (500 MHz, DMSO-d_6). **b)** ^{13}C NMR spectrum of **4b** (100 MHz, DMSO-d_6).

a

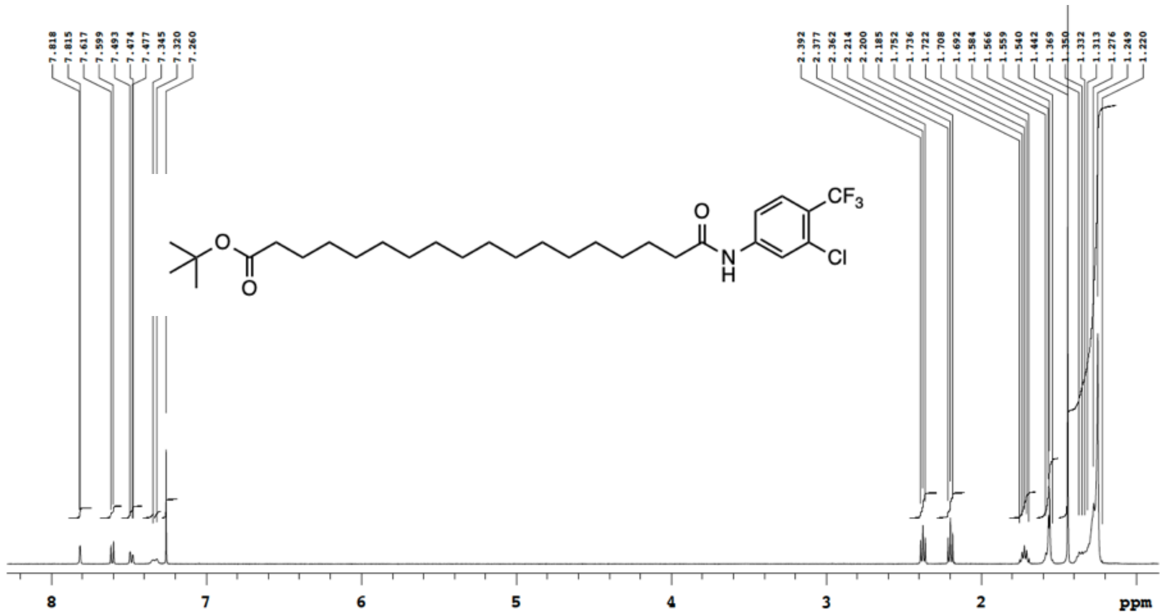

b

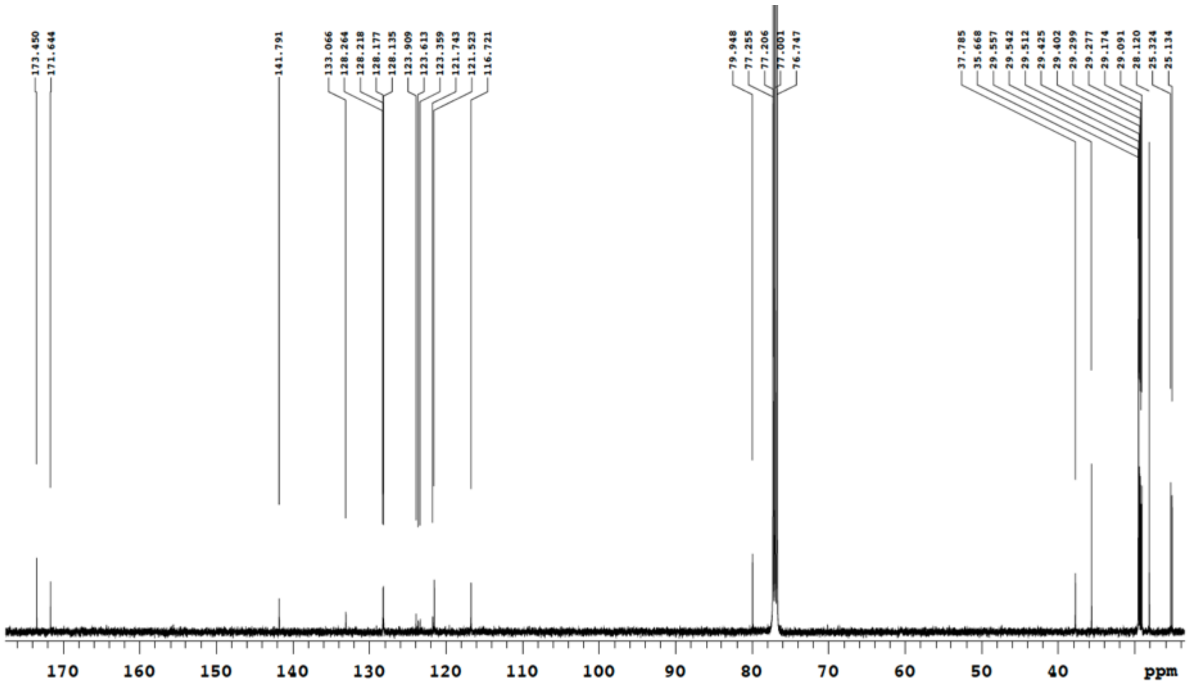

Figure S30: a) ^{1}H NMR spectrum of **5a** (400 MHz, $CDCl_3$). **b)** ^{13}C NMR spectrum of **5a** (125 MHz, $CDCl_3$).

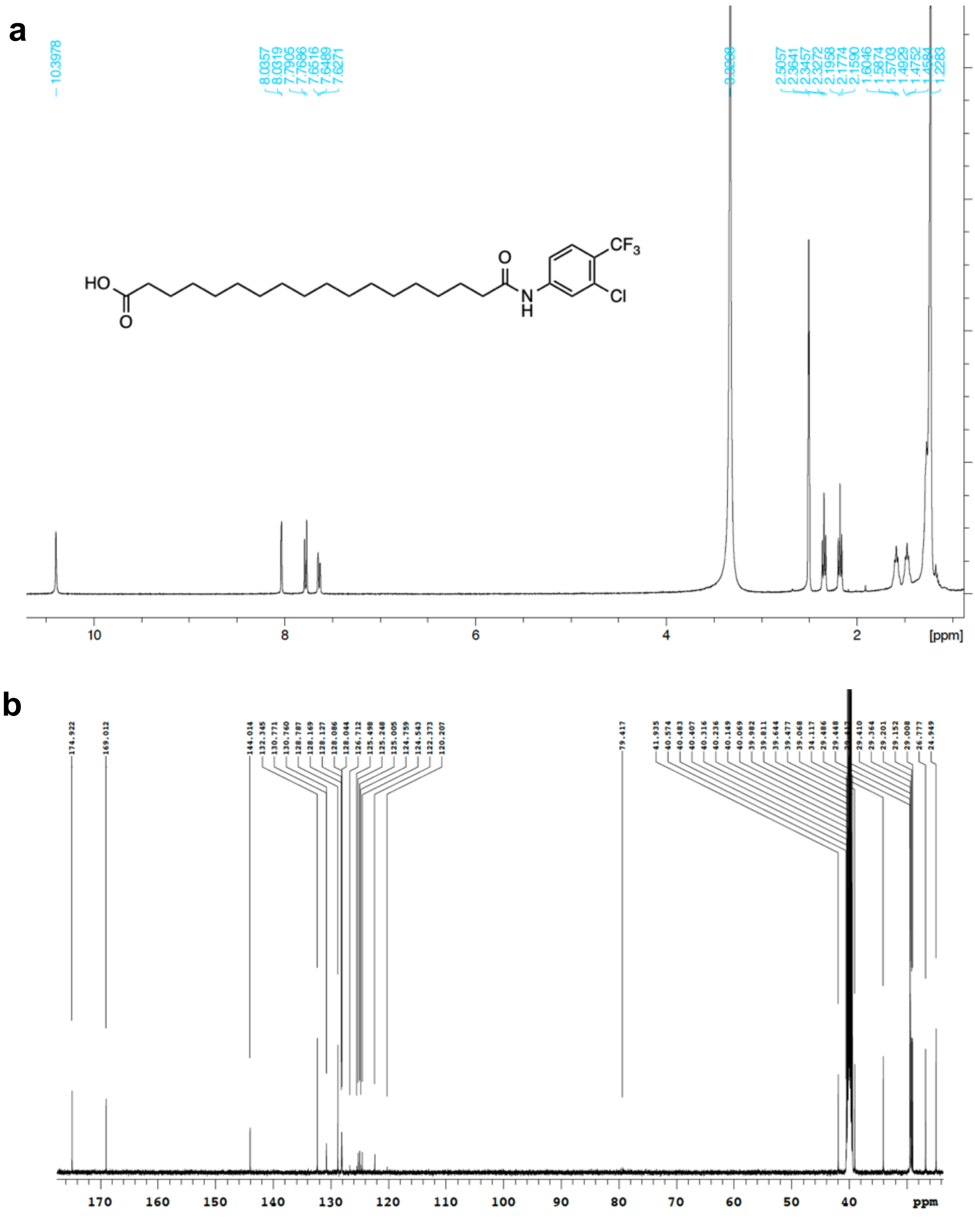

Figure S31: a) ^{1}H NMR spectrum of **5b** (500 MHz, DMSO-d_6). **b)** ^{13}C NMR spectrum of **5b** (125 MHz, DMSO-d_6).

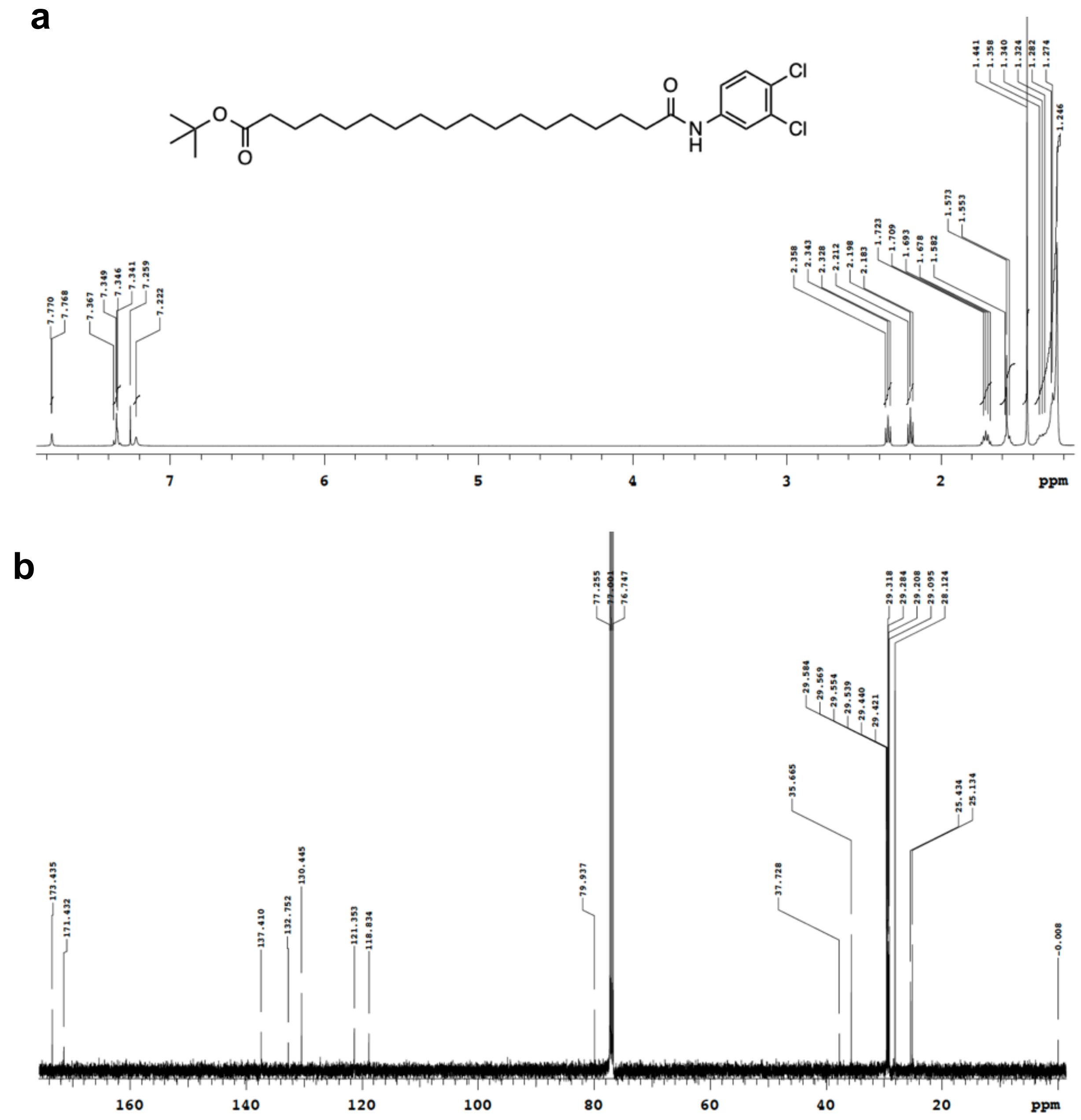

Figure S32: a) ^{1}H NMR spectrum of **6a** (400 MHz, $CDCl_3$). **b)** ^{13}C NMR spectrum of **6a** (125 MHz, $CDCl_3$).

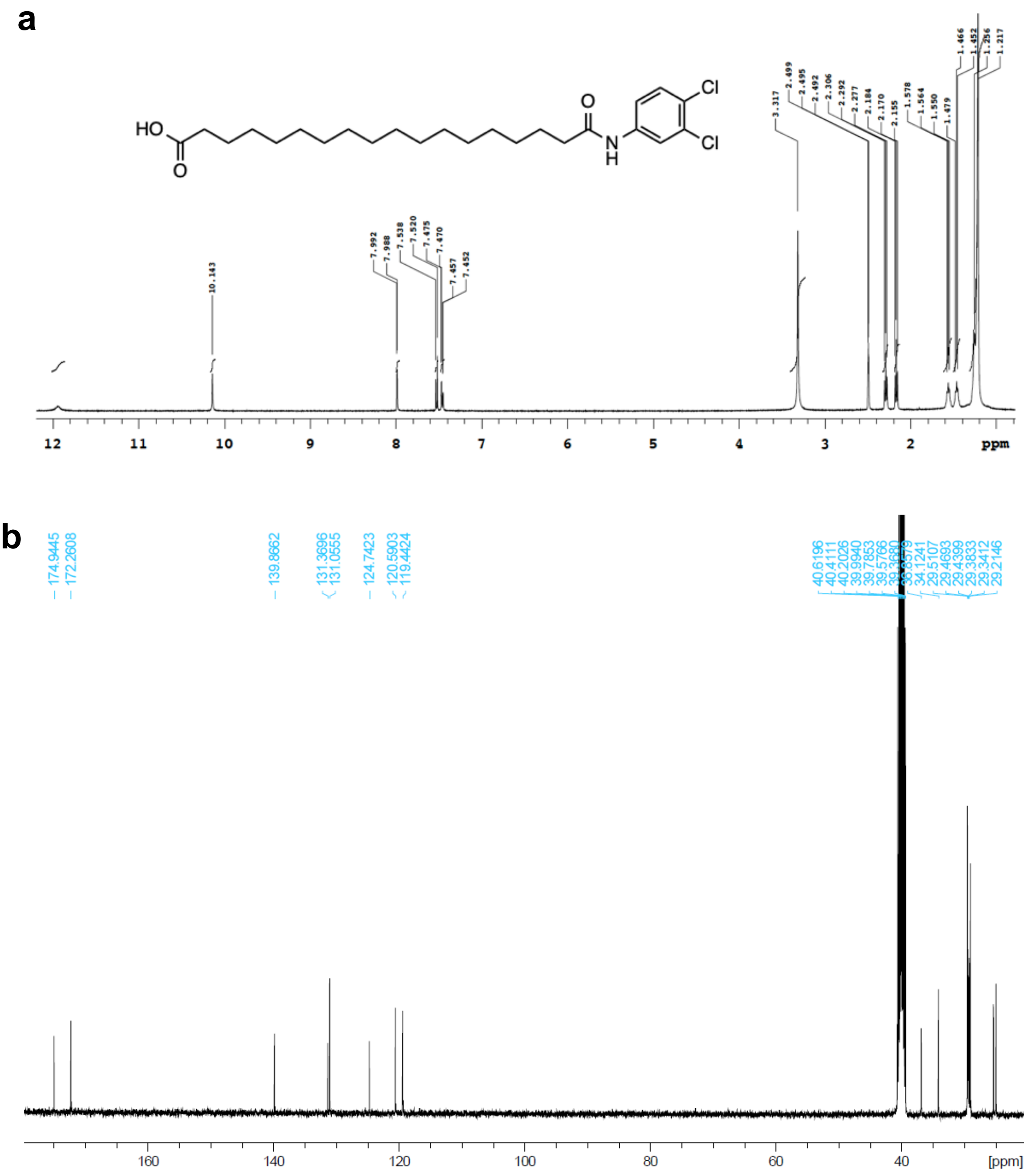

Figure S33: a) ^{1}H NMR spectrum of **6b** (500 MHz, DMSO-d_6). **b)** ^{13}C NMR spectrum of **6b** (100 MHz, DMSO-d_6).

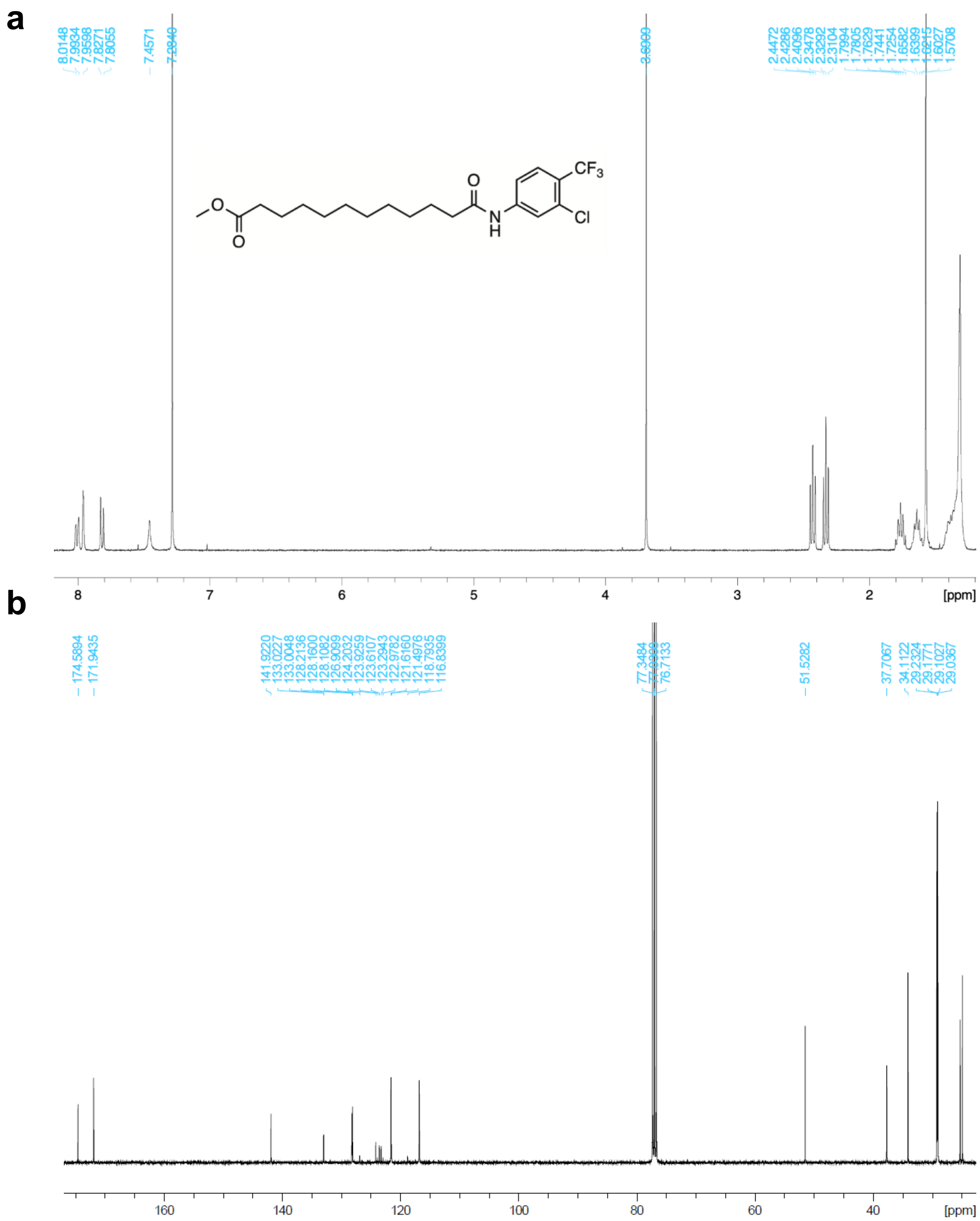

Figure S34: a) ^{1}H NMR spectrum of **5a-C12** (400 MHz, $CDCl_3$). **b)** ^{13}C NMR spectrum of **5a-C12** (100 MHz, $CDCl_3$).

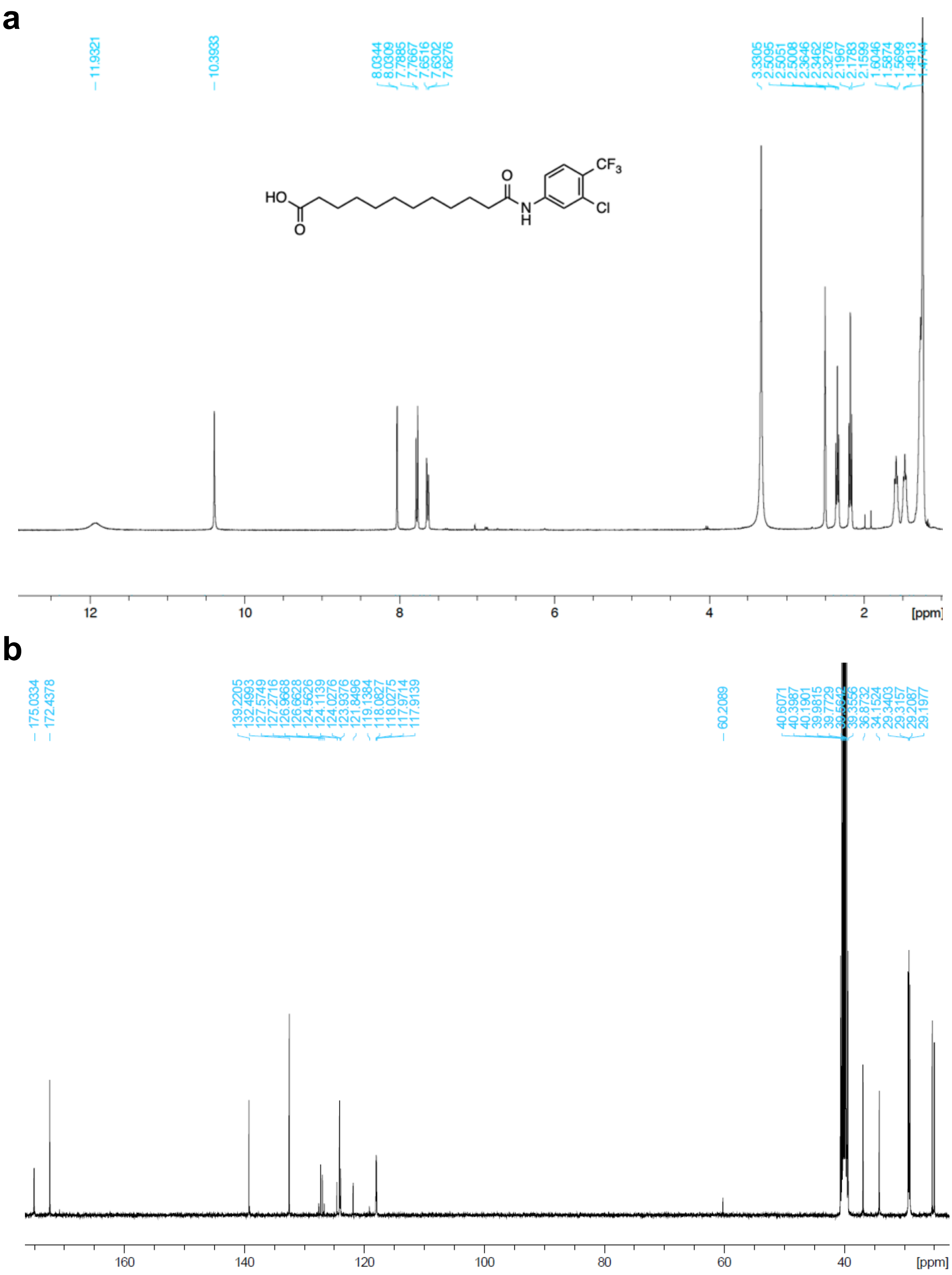

Figure S35: a) ^{1}H NMR spectrum of **5b-C12** (500 MHz, DMSO-d_6). **b)** ^{13}C NMR spectrum of **5b-C12** (100 MHz, DMSO-d_6).

13. References

1. C. Hansch, A. Leo and R. W. Taft, *Chem Rev*, 1991, **91**, 165-195.
2. I. V. Tetko and V. Y. Tanchuk, *J Chem Inf Comput Sci*, 2002, **42**, 1136-1145.
3. X. Wu and P. A. Gale, *J Am Chem Soc*, 2016, **138**, 16508-16514.
4. X. Wu, P. Wang, W. Lewis, Y.-B. Jiang and P. A. Gale, *Nat Commun*, 2022, **13**.
5. D. Brynn Hibbert and P. Thordarson, *Chem Commun*, 2016, **52**, 12792-12805.
6. P. Thordarson, Bindfit, http://supramolecular.org, (accessed 12/07/2023, 2023).
7. R. K. Dennington, Todd A.; Millam, John M. Semichem *Journal*, 2016.
8. M. J. Frisch, G. W. Trucks, H. B. Schlegel, G. E. Scuseria, M. A. Robb, J. R. Cheeseman, G. Scalmani, V. Barone, G. A. Petersson, H. Nakatsuji, X. Li, M. Caricato, A. V. Marenich, J. Bloino, B. G. Janesko, R. Gomperts, B. Mennucci, H. P. Hratchian, J. V. Ortiz, A. F. Izmaylov, J. L. Sonnenberg, Williams, F. Ding, F. Lipparini, F. Egidi, J. Goings, B. Peng, A. Petrone, T. Henderson, D. Ranasinghe, V. G. Zakrzewski, J. Gao, N. Rega, G. Zheng, W. Liang, M. Hada, M. Ehara, K. Toyota, R. Fukuda, J. Hasegawa, M. Ishida, T. Nakajima, Y. Honda, O. Kitao, H. Nakai, T. Vreven, K. Throssell, J. A. Montgomery Jr., J. E. Peralta, F. Ogliaro, M. J. Bearpark, J. J. Heyd, E. N. Brothers, K. N. Kudin, V. N. Staroverov, T. A. Keith, R. Kobayashi, J. Normand, K. Raghavachari, A. P. Rendell, J. C. Burant, S. S. Iyengar, J. Tomasi, M. Cossi, J. M. Millam, M. Klene, C. Adamo, R. Cammi, J. W. Ochterski, R. L. Martin, K. Morokuma, O. Farkas, J. B. Foresman and D. J. Fox, Gaussian Rev C01, 2016.
